# Impact of Educational Intervention on Hygiene Knowledge and Practices of Sanitation Workers Globally: A Systematic Review

**DOI:** 10.1155/sci5/3265559

**Published:** 2025-08-25

**Authors:** Alive Ntunja, Wilma Ten-Ham Baloyi, June Teare, Oyedele Opeoluwa, Paula Melariri

**Affiliations:** ^1^Department of Environmental Health, Nelson Mandela University, Gqeberha, South Africa; ^2^Department of Nursing Science, Nelson Mandela University, Gqeberha, South Africa; ^3^Environment and Health Research Unit, South African Medical Research Council, Johannesburg, South Africa; ^4^Department of Computing, Mathematical and Statistical Sciences, School of Science, University of Namibia, Windhoek, Namibia

**Keywords:** educational programs, hygiene knowledge, impact, practices, predictor factors, sanitation workers

## Abstract

**Background:** Sanitation workers are also known as ‘garbage workers' who play a significant role in the sanitation chain. For many generations, sanitation workers' level of knowledge regarding hygiene practices remains low due to a lack of educational programs on hygiene. As a result, they are widely exposed to hygiene-related diseases such as cholera, skin infections and various other diseases, increasing their risk of mortality to 40%. This review aims to explore the global impact of educational programs on the hygiene knowledge and practices of sanitation workers.

**Methods:** The systematic literature search was conducted for studies published between 2013 and 2023 using the following databases: MEDLINE (via EBSCOHost), PubMed and Google Scholar to identify quantitative studies on the subject. Study quality was assessed using the Joanna Briggs Institute Critical Evaluation Instruments. Data extracted from the included articles were presented using a summary of findings table and presented graphically through charts and tables, employing both descriptive and inferential statistical methods. The PRISMA flow diagram was used to present the article selection process.

**Results:** The systematic review included 15 eligible studies from a total of 2777 articles. At least 60% (*n* = 9) of the reviewed studies found educational program relating to hygiene to have a positive impact on sanitation workers' hygiene knowledge and practices. However, most (*n* = 7) studies indicated that the efficacy of programs on hygiene knowledge and practices is indirectly influenced by educational background, age and work experience (predictor factors).

**Conclusion:** Educational programs regarding hygiene have the potential to significantly improve sanitation workers knowledge and practices. Findings also suggest the implementation of active and intensive intervention programs to improve sanitation workers hygiene knowledge and practices.

## 1. Introduction

Hygiene practices are a set of behaviours that can improve cleanliness and minimise the spread of infectious diseases [1]. Sanitation refers to the provision of facilities or services that protect people from their urine and faeces [2]. Subsequently, sanitation is the provision of clean and healthy environment. Adequate sanitations are crucial needs for human health, which reduce the transmission of neglected tropical diseases (NTDs) such as diarrhoea, leprosy, stunted development in children and other ailments [3]. Lack of hygiene practices and inadequate sanitary conditions have a devastating impact on the increased burden of communicable diseases in developing countries, such as South Africa, Nigeria and India [4].

According to Fogang et al. [5], the lack of safe hygiene practices threatens the lives of millions of people daily. Unsafe hygiene practices are important determinants of several infectious diseases such as diarrhoea, cholera and dysentery [6]. Over 775,000 deaths globally are recorded each year due to unsafe hygiene practices [7]. In most developing countries, an increased mortality rate of 11% among children under 5 years of age, resulting from diarrhoeal cases, is due to hygiene-related challenges [8]. Globally, it is estimated that over 889,000 deaths are attributed to communicable diseases, which are largely caused by poor hygiene practices [8]. Globally, 7% of the global burden of diseases and 19% of child mortalities are associated with inadequate sanitation and lack of hygiene practices [9].

Poorly managed domestic wastes eventually return to the natural environment and create serious environmental degradation and pollution [10]. Furthermore, improperly managed domestic wastes have been linked to unhygienic living conditions and various infectious diseases that are spread or transmitted by disease vectors such as mosquitoes and insects harboured by such conditions in the communities [11]. It is, therefore, vital to ensure that the environment is protected from becoming degraded or polluted. Sanitation workers, often referred to as ‘garbage workers,' play a crucial role in the sanitation chain, which spans from waste collection and transportation to waste disposal at dumping sites. This chain also includes street sweepers, latrine cleaners and waste transporters [12]. These workers occupy a key position in providing a public service essential for ensuring adequate sanitation and hygiene for all [13].

Sanitation workers are responsible for collecting waste materials at their source, clearing clogged sewers, emptying trash cans and transporting waste to designated disposal facilities [14]. However, there is a significant lack of hygienic awareness and practices among this group, which is vital for their safety and that of the community while performing their duties [15]. Zolnikov et al. [16] highlighted that sanitation workers are a vulnerable population due to their low levels of education, which limits their opportunities for more suitable employment. Studies have shown that for many generations, sanitation workers have had low levels of hygiene knowledge due to insufficient educational intervention programs [17–19]. Philippe et al. [13] noted that sanitation workers often lack knowledge about the proper use and disposal of Personal Protective Equipment (PPE), such as gloves and face masks. A cross-sectional study among 576 sanitation workers in Ethiopia found that only 19% (109/576) had received formal training in hygiene practices, resulting in 89% (513/576) being unaware of the importance of appropriate PPE [20]. This finding is supported by a descriptive study conducted in Maggarai Regency, which reported that 100% of the sanitation workers did not use complete PPE due to a lack of awareness and knowledge about good sanitation and hygiene practices [21].

Research has shown that poor hygiene knowledge and practices among sanitation workers are highly correlated with hygiene-related diseases such as cholera, dysentery, taeniasis and diarrhoea [20, 22]. Poorly managed domestic waste can lead to severe environmental degradation and pollution, creating unhygienic conditions that foster disease vectors like mosquitoes and insects, thereby spreading infectious diseases [10, 11]. Sanitation workers are also widely exposed to diseases such as leptospirosis, occupational asthma, lung function abnormalities and skin infections, increasing their mortality risk by 40% [23]. In the United States, annually, at least 180 out of every 1000 sanitation workers are at risk of hygiene-related infections or injuries [24]. In sub-Saharan Africa, more than 54,000 sanitation workers die each year due to work-related diseases and injuries exacerbated by inadequate hygiene training [25].

Data on the correlation between hygiene-related knowledge, practices and educational training programs among sanitation workers are crucial for predicting and addressing infectious disease proportions. This information aids in strategic policy-making for public health resource allocation [26]. Despite a systematic review by Ricci et al. [27] focussing on behavioural changes in sanitation workers, there has been no comprehensive review on this topic. Therefore, this systematic review aims to determine the impact of educational programs on sanitation workers' hygiene knowledge and practices globally.

## 2. Methods

### 2.1. Protocol and Registration

The review was guided by the Preferred Reporting Item for Systematic Review and Meta-Analysis (PRISMA). The review involved a protocol registration on PROSPERO (Prospective Register of Systematic Reviews) database (Identifier: CRD42023466319). The first author conducted each step of the systematic review, including the search of articles (peer-reviewed articles), screening, data extraction and synthesis, risk appraisal, report writing following PRISMA (2020) guidelines and publication.

### 2.2. Review Question

The review question guiding the search strategy was formulated using the Population, Intervention, Comparison, and Outcome (PICO) framework as follows: What is the impact of educational programs on improving sanitation workers' hygiene-related knowledge and practices?

  Population: sanitation workers
  Intervention: education programmes
  Comparison: no educational program
  Outcome: enhanced hygiene knowledge and practices

### 2.3. Search Strategy

The systematic search (see Annexure S1), conducted by A.N. in March 2024, reviewed studies from 2013 to 2023 using EBSCOHost (MEDLINE), PubMed and Google Scholar. Key terms included ‘sanitation worker^∗^ OR waste collector^∗^ OR garbage collectors^∗^ AND hygiene knowledge OR practice^∗^ AND educational programs^∗^ OR training programs.' Data management, citation generation and literature storage were handled using Mendeley. Boolean operators and truncation were employed with librarian assistance, and secondary articles were identified through reference searching and screening.

### 2.4. Selection Process and Eligibility Criteria

Studies were selected through a two-step review process. First, reviewers screened titles and abstracts to identify relevant articles. Second, they reviewed the full texts (see Table S2). Disagreements were resolved through discussion, with a third reviewer. Eligible studies included peer-reviewed quantitative articles published in English from 2013 to 2023. Exclusions included studies not focussing on hygiene knowledge and practices or educational programs for sanitation workers, as well as duplicates and grey literature.

### 2.5. Critical Appraisal

The review's findings were assessed for reliability, validity and applicability using the Joanna Briggs Institute (JBI) tool for critical appraisal of analytical cross-sectional studies (see Checklist S3). The evaluation criteria included clear inclusion criteria, detailed population and setting descriptions, valid exposure measurement, objective and standard criteria, identification and management of confounding factors, valid outcome measurement and appropriate statistical analysis. Three reviewers independently evaluated the studies, with conflicts resolved by a fourth reviewer. The PRISMA checklists (see Tables S4 and S5) were used to ensure transparency. A scoring system was employed where ‘No,' ‘Unclear' and ‘Not applicable' received a score of 0, and ‘Yes' received a score of 1. Scores were converted to percentages and categorized as low rigour (0%–59%), medium rigour (60%–79%) or high rigour (80%–100%). Only studies with medium and high rigour were included.

### 2.6. Data Extraction and Analysis

**Figure 1:**
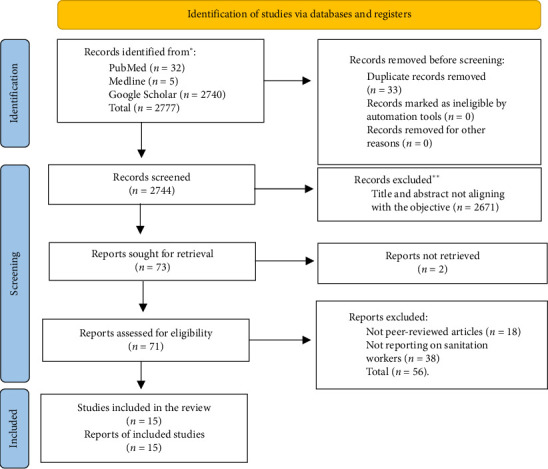
PRISMA (2020) flow diagram summarizing the study selection.

Data extracted from the 15 reviewed studies were summarized in a table created by A.N., capturing details such as author/year, study location, study type, sanitation worker category, educational level, institutional affiliation, intervention method, pre- and postintervention periods and main findings. The PRISMA (2020) flowchart (Figure 1) illustrated the study selection process. The extracted data from the reviewed studies were synthesized using a thematic analysis. In this analysis, the data were reviewed repeatedly, codes were developed and themes were identified. Since some of the reviewed studies did not provide standard deviation values in their studies, it became quite impossible to perform a meta-analysis on the pre–post mean values. For this reason, the extracted data were qualitatively analysed using the descriptive statistics approach.

### 2.7. Ethics

This systematic review did not involve human participants, relying solely on publicly available data from peer-reviewed articles. The integrity of the research was maintained through honesty and transparency in the reporting processes, and plagiarism was strictly avoided.

## 3. Results

### 3.1. Search and Selection of Studies' Results

The systematic review identified articles on the impact of educational programs on sanitation workers' hygiene-related knowledge and practices. The search retrieved a total of 2777 studies. After removing 33 duplicates, 2744 articles remained for relevance screening based on titles and abstracts. Two reviewers selected 71 articles for full-text review. This assessment resulted in the exclusion of 2 articles that were not retrieved and 2671 articles that did not meet the indicated criteria: (1) titles and abstracts not aligning with the objective and (2) not being peer-reviewed articles. Finally, 15 studies were retained after the full-text review, with 56 articles excluded for not meeting the study requirements or inclusion criteria. Figure 1 shows the PRISMA flow diagram that summarizes the selection and exclusion process during the systematic search of the study. The retained 15 studies were further appraised, with 3 scoring 63% (medium rigour), 6 scoring 75% (medium rigour), 4 scoring 87.5% (high rigour) and 2 scoring 100% (high rigour). All 15 articles were included in the review.

### 3.2. Study Characteristics

The retained 15 studies included in this review are summarized in Table 1. The table provides details on the author/year of the study, study design, study location, study aims, category of sanitation worker, most common educational level of the studied population, institutional affiliation, intervention method used, period gap between pre- and posteducational program and study conclusions.

**Figure 2:**
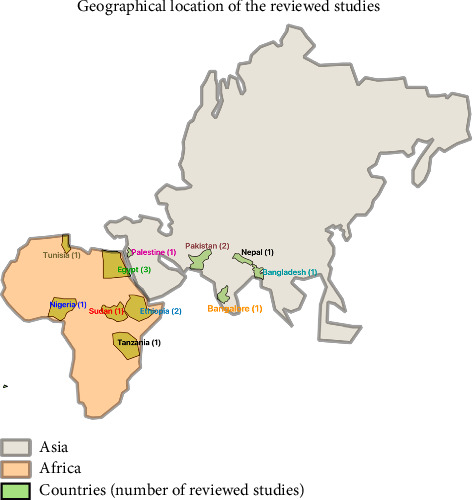
Map showing the geographical location of reviewed studies (*Source:* The map was generated by the first author using QGIS 3.36 Maidenhead).

The reviewed studies primarily originated from low-income countries, including Sudan (*n* = 1), Pakistan (*n* = 2), Bangladesh (*n* = 1), Bangalore (*n* = 1), Nepal (*n* = 1), Palestine (*n* = 1), Southern Tunisia (*n* = 1), Ethiopia (*n* = 2), Egypt (*n* = 3), Tanzania (*n* = 1) and Nigeria (*n* = 1). The geographical locations of these studies are illustrated in Figure 2.

Furthermore, 60% of the 15 reviewed studies used a quasiexperimental study design to evaluate the impact of educational interventions on sanitation workers' hygiene knowledge and practices in their respective study [29–31, 34–39]. The remaining 40% used a cross-sectional study design in their respective study [20, 24, 25, 28, 32, 33].

### 3.3. Studied Population

The included studies encompassed different categories of sanitation workers within the sanitation chain, ranging from points of waste collection, transportation and up to disposal sites. These sanitation workers included municipal solid waste workers (MSWWs), garbage collectors, street sweepers, sewage workers and healthcare sanitary staff.

**Figure 3:**
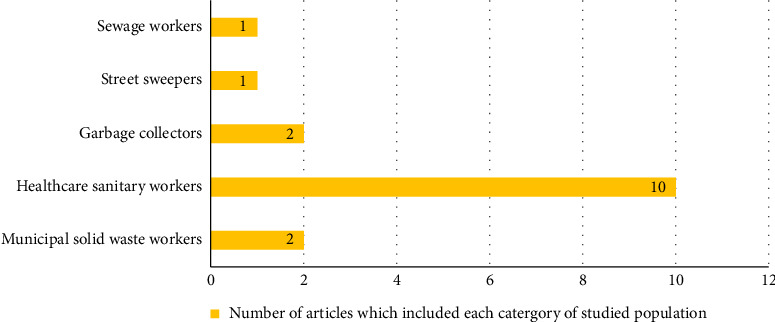
The most frequently studied categories of sanitation workers.

The most extensively researched category of sanitation workers was healthcare sanitary staff, featured in 67% (10/15) of the reviewed studies. This was followed by garbage collectors and MSWWs, each appearing in 2 studies (27%). Street sweepers and sewage workers were represented in only 1 study each (7%) [34, 36].  Figure 3 illustrates the most frequently studied categories of sanitation workers in the review.

**Figure 4:**
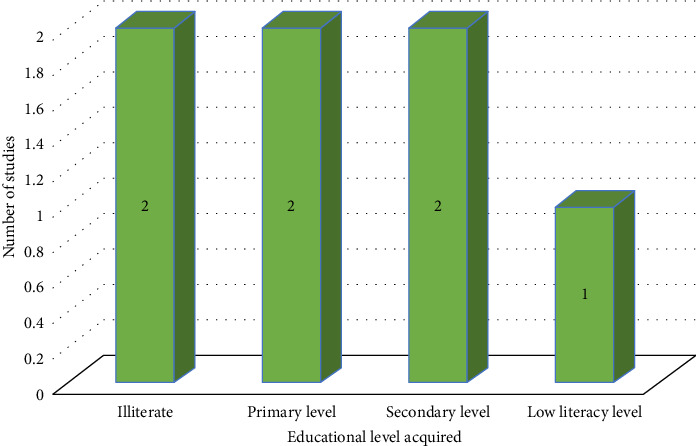
The educational level acquired by the majority of sanitation workers per study.

More than 47% (7/15) of the reviewed studies underscored that the majority of sanitation workers have attained a low educational level [20, 25, 32–34, 36, 38] (refer to Figure 4). Within these studies, 29% (2/7) revealed that the majority of sanitation workers were illiterate (refer to Table 1) [25, 34], while another 29% (2/7) reported that they had only completed primary education (grades 1–7) [20, 33]. Awad et al. [38] found that a significant proportion (14%) of sanitation workers in their study had basic literacy skills restricted to reading and writing abilities only. The highest educational level attained by the majority of the studied population in the reviewed studies was secondary education (grades 8–12) [32, 36]. The educational levels achieved by the majority of sanitation workers in each study are depicted in Figure 4.

In 33% (5/15) of the reviewed studies [20, 25, 34, 36, 38], sanitation workers were associated with municipal institutions. Conversely, in 77% (10/15) of the reviewed studies [24, 28–33, 35, 37, 39], the studied population was linked with healthcare facilities, including tertiary hospitals, government hospitals and private hospitals.

### 3.4. Educational Program

**Figure 5:**
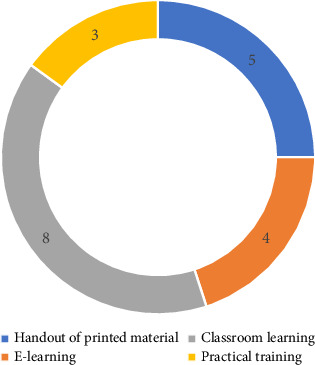
Frequently used intervention methods in the reviewed studies.

Educational programs, one of the variables evaluated in this review, were a consistent variable across all 15 studies reviewed. However, only 9 (60%) studies provided relevant information on the implemented intervention programs to assess their impact on participants' hygiene-related knowledge and practices [29–31, 34–39]. Conversely, the remaining 6 (40%) studies solely discussed the significance of implementing educational programs on hygiene-related knowledge and practices, as well as their role in the prevention of hygiene-related diseases [20, 24, 25, 28, 32, 33]. Regarding the training or intervention methods used (refer to Figure 5), they can be categorized as follows:

⁃ Handout of printed material (*n* = 5): pamphlets, flyers, posters and brochures that presented hygiene-related information on safe waste handling.
⁃ E-learning (*n* = 4): the education delivered online through the use of any digital devices.
⁃ Classroom learning (*n* = 8): theory lessons (guidelines and regulations) regarding hygiene and focus group discussions.
⁃ Practical training (*n* = 3): education delivered through demonstrations.

Figure 5 shows the frequently used intervention methods in the reviewed studies.

The trainer responsible for implementing these intervention methods in a prearranged venue, aimed at enhancing sanitation workers' hygiene-related knowledge and practices during their duties, was either a primary investigator (researcher) or the research team [29, 31, 34–39].

In 8 out of the 9 studies that provided pertinent information on the implemented program, the research team was responsible for implementing the intervention method [29, 31, 34–39].

The period gap between the implementation of the intervention method and the postassessment of hygiene knowledge and practices among sanitation workers was monitored in 53% (8/15) of the reviewed studies. Out of the 9 studies, only 2 (22%) allowed a period gap of 1 month between the intervention phase and the post-test phase [34, 35]. Four studies (44%) allowed a period gap of 3 months [29, 36, 37, 39], one study (11%) allowed a period gap of 6 months [31] and one study (13%) allowed a 2-month period gap [38] (see Table 2).

### 3.5. Impact of Educational Program

Results from 15 studies were thoroughly examined, and published findings regarding the impact of educational programs on sanitation workers' hygiene knowledge and practice were retrieved. To mitigate bias, interpretations in this review were grounded solely on empirical estimates rather than the authors' subjective interpretation of their results.

**Figure 6:**
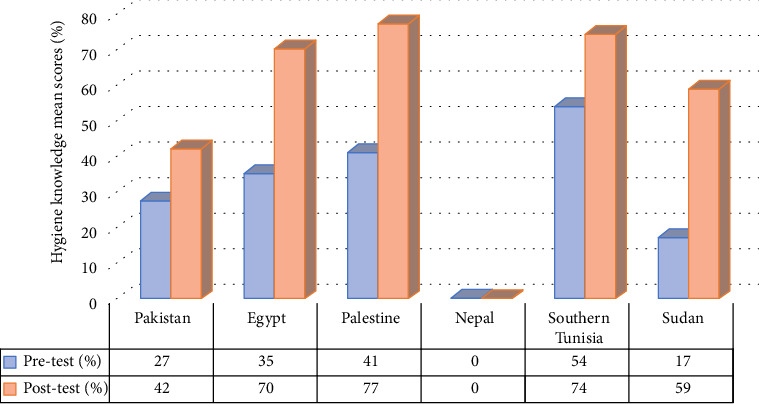
Mean scores of hygiene-related knowledge pre and post an educational program by country.

**Figure 7:**
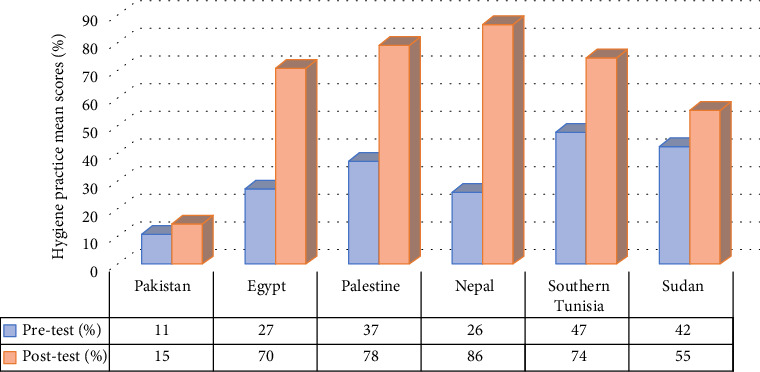
Mean scores of hygiene-related practice pre and post an educational program by country.

In most of the reviewed studies, a significant increase in the average mean scores of hygiene knowledge and practice among sanitation workers' posteducational programs, compared with the baseline data, was evident. Out of the 15 reviewed studies, only 9 studies (conducted in Pakistan [*n* = 2], Egypt [*n* = 3], Nepal [*n* = 1], Palestine [*n* = 1], Southern Tunisia [*n* = 1] and Sudan [*n* = 1]) provided hygiene knowledge and practice mean scores of sanitation workers' pre- and postintervention programmes. The mean scores of baseline data on hygiene-related knowledge and practice, compared with post-test mean scores by country, are depicted in Figures 6 and 7.

Table 3 presents the pre- and postintervention hygiene-related knowledge and practice mean scores along with their standard deviations (SDs) observed in the 9 studies selected for this review. However, the study by Sapkota et al. [30] did not report the pre- and postintervention mean scores on hygiene knowledge due to its sole focus on evaluating the impact of educational intervention on the practices of the studied population. Similarly, Kumar et al. [39] did not report on the pre- and postintervention mean scores on hygiene-related practice in their study as it primarily aimed to evaluate the impact of educational intervention on hygiene knowledge alone. Consequently, Sapkota et al. [30] was excluded from the knowledge section of the table, while Kumar et al. [39] was excluded from the practice section.

Upon pooling the pre- and postintervention mean scores, a positive improvement was noted in the participants' hygiene knowledge scores following the educational intervention, with improvement values ranging from 0.7% to 43%. Similarly, a positive improvement was observed in the participants' hygiene practice scores postintervention, with improvement values ranging from 4% to 61%.

The other 6 studies mainly provided a pertinent information on the significance of implementing intervention program among the studied population. In 67% (10/15) of the reviewed studies, it was evident that the lack of intervention programs had a negative impact on the transmission of hygiene-related diseases such as cholera, hepatitis B and C, tuberculous (TB), respiratory infections, skin lesions and various other diseases [20, 24, 25, 30, 32, 34–36, 38, 39].

### 3.6. Predictor Factors of Hygiene Knowledge and Practices Amongst Sanitation Workers Post an Educational Program

The following three predictor factors: (1) educational level acquired, (2) age and (3) work experience of sanitation workers were independently associated with an increasing hygiene knowledge and practice change posteducational program.

### 3.7. Educational Level

Seven out of 15 reviewed studies [24, 25, 33–36] demonstrated a substantial positive impact on sanitation workers' hygiene knowledge and practice, particularly among those with formal education. This improvement was observed in pre- to postintervention tests and was more significant compared with workers with limited literacy skills.

### 3.8. Age

Two (13%) out of 15 reviewed studies [35, 36] found a significant inverse relationship between the age of the studied population and improvements in hygiene-related knowledge and practice prior to and following the intervention program, assuming all other parameters are constant.

### 3.9. Work Experience

**Figure 8:**
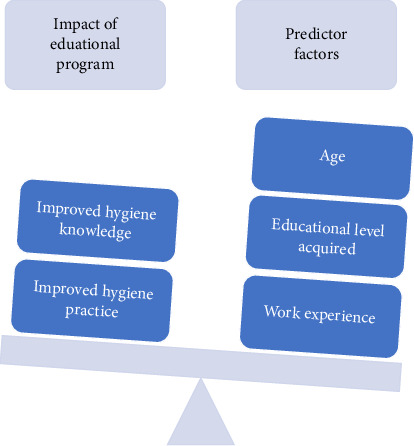
The impact predictor factors have on the effectiveness of the intervention program on sanitation workers' hygiene knowledge and practice.

Finally, 2 studies [35, 36] show that the effectiveness of educational program is significantly influenced by individuals' work experience.  Figure 8 illustrates the expected impact these predictor factors have on the effectiveness of intervention program on sanitation workers' hygiene knowledge and practice change.

## 4. Discussion

This systematic review aimed to assess the global impact of educational programs on sanitation workers' hygiene knowledge and practices. To the best of our knowledge, this is the first systematic review conducted on this subject. Articles meeting our inclusion criteria contributed to a significant body of evidence, all originating from low-income countries. None of the reviewed studies were from high-income countries. We evaluated three key variables: hygiene knowledge, hygiene practice and the effectiveness of educational programs.

The findings of the current systematic review underscored the unsatisfactory state of hygiene knowledge and practices among sanitation workers' preintervention programs. This deficiency likely stemmed from a dearth of hygiene-focused training programs in their work environments. For instance, a study in Sudan by Melaku and Tiruneh [20] found that over 73% of the sanitation workers did not practice personal hygiene after handling waste due to this lack of training. Furthermore, Shoaib et al. [40] also revealed that only 20% (70/347) sanitation workers in Bangladesh ever received hygiene-related training programs. Consequently, they faced heightened risks of various hygiene-related diseases, including diarrhoea, cholera and hepatitis B, as documented by Sarker et al. [24], Kumar et al. [39], Onoh et al. [32], Melaku and Tiruneh [20], AbouZeid et al. [34], Awad et al. [38], Ben Jmaa et al. [35], Mohamed and Mohamed [36] and Temesgen et al. [25]. In addition, Sapkota et al. [30] emphasized that inadequate hygiene knowledge and practices during waste handling could facilitate the transmission of more than 30 hygiene-related pathogens. Ayilara et al. [11] further indicated that improperly managed domestic waste has been linked to unhygienic living conditions and various infectious diseases that are spread or transmitted by disease vectors such as mosquitoes and insects harboured by such conditions in the communities.

The results of the systematic review clearly demonstrate a marked improvement in hygiene knowledge and practice mean scores following the implementation of educational programs. This positive impact underscores the effectiveness of such initiatives in enhancing sanitation workers' understanding and application of hygiene principles. Specifically, the study revealed a noteworthy enhancement in participants' hygiene knowledge scores postintervention, ranging from 0.7% to 43%, alongside improvements in hygiene practice scores ranging from 4% to 61%. Robat et al. [41] further revealed a significant improvement in hygiene knowledge (*p* < 0.001) and practices (*p*=0.001) of healthcare sanitation workers' posteducational program. In other words, the implemented educational intervention had a notable impact on participants' hygiene knowledge and practices. Nevertheless, the study done in Pakistan revealed contradictory findings, indicating that the hygiene knowledge and practices of healthcare sanitary workers actually deteriorated after participating in a training program [35]. This may be attributed to the absence of Supporting information through educational materials, such as posters and flyers, during the intervention phase. However, it is important to recognize that the efficacy of these educational programs is contingent upon several contributing factors, including the type of intervention method employed, the age of participants, their work experience and educational background [35, 39]. These variables play a pivotal role in shaping the outcomes of hygiene education initiatives, underscoring the need for tailored approaches to address the diverse needs of sanitation workers.

In the majority of the reviewed studies providing details on implemented education programs, these initiatives were crafted by research teams drawing from existing literature and national cleaning guidelines and policies [29, 31, 34–39]. Among these studies, classroom learning emerged as the most commonly used intervention method, accounting for over 53% (6/15) of the cases. This approach typically involved theoretical instruction on hygiene-related international guidelines and regulations, supplemented by interactive focus group discussions [29–31, 34, 36, 37].

Following closely behind, printed materials such as flyers, brochures and posters were also frequently employed to disseminate hygiene-related messages to sanitation workers [30, 31, 34, 35, 38]. The widespread adoption of these intervention methods can be attributed to their cost-effectiveness and ease of implementation. However, it is crucial to note that the choice of intervention method significantly influences the effectiveness of educational programs in improving sanitation workers' knowledge and practices. This notion finds support in the research of Kumar et al. [29], who observed that hygiene-related educational programs yield greater efficacy when complemented by innovative approaches such as extended trainings and physical demonstrations.

Similar conclusions were drawn from a study conducted in Southern Tunisia, which highlighted the substantial impact of intensive and periodic refresher training methods on enhancing hygiene practices among sanitation workers [35]. These findings underscore the importance of employing multifaceted approaches tailored to the specific needs and contexts of sanitation workers to achieve meaningful improvements in hygiene knowledge and practices.

In 60% (9/15) of the studies reviewed, a 3-month period gap was implemented between the intervention phase and the post-test phase to mitigate courtesy bias among participants during the postassessment [29–31, 34–39]. However, this systematic review indicates there is no statistically significant difference (*p* > 0.05) in the impact of the educational program on the hygiene knowledge and practices of sanitation workers, regardless of the duration of the period gap.

This lack of significant difference may stem from the absence of reinforcing or additional hygiene-related information provided during the period gap between the intervention phase and the final follow-up in most pre–post-test studies. Elnour et al. [37] supported this observation, the finding that the period gap between the intervention phase and the postassessment did not influence the improvement of participants' hygiene knowledge and practice. This suggests that while implementing a period gap can help minimize courtesy bias, its effectiveness in enhancing the outcomes of educational programs for sanitation workers may be limited without supplementary interventions during this time frame.

In this systematic review study, as previously discussed in the result section, it is further evident that predictor factors such as educational background, age and work experience acquired by sanitation workers play a significant role in the impact educational programs have on their hygiene knowledge and practices [35, 36]. This is contradictory to the findings by Kumar et al. [39] that there is no statistically significant association between satisfactory hygiene knowledge and practices, age and work experience post educational program.

Ben Jmaa et al. [35] highlighted a significant gap in the improvement of hygiene knowledge and practices between medical practitioners and sanitation workers after participating in an educational program, revealing a notable disparity in the effectiveness of the program across different educational backgrounds. This discrepancy may stem from variations in educational backgrounds and fundamental technical skills within the studied cohorts. Such findings align with research conducted in Ethiopia, which revealed that posteducational program garbage workers with a secondary education level exhibited significantly better hygiene-related practices compared with their illiterate counterparts [42].

Regarding the age of sanitation workers, it has been established that as individuals age, the effectiveness of training programs on hygiene knowledge and practices diminishes [35]. This decline may be attributed to age-related cognitive decline, making it less likely for older workers to retain and implement the information conveyed during intervention programs. Consistent with this, a study in North India found that workers aged 20–30 years demonstrated significantly higher knowledge following educational programs compared with middle-aged workers (41–59 years old) [43]. However, this contradicts the findings of Mohamed and Mohamed [36], who observed that sewage workers older than 40 years exhibited significantly better mean hygiene knowledge and behaviour scores post training.

Concerning work experience, reviewed studies indicate that the more experienced sanitation workers are, the less likely they are to experience significant positive changes in knowledge and practices following educational programs [36]. This may be attributed to the entrenched nature of existing knowledge and practices among experienced workers. However, contradictory findings were noted in a systematic review by Ricci et al. [27], suggesting that sanitation workers with greater work experience possess a greater ability to adopt new behaviours or attitudes, drawing from their existing experiences within their occupational context.

## 5. Conclusion

The findings from this systematic review underscore the positive impact of educational programs on enhancing the hygiene knowledge and practices of sanitation workers. However, certain demographic factors such as educational background, age and work experience emerged as potential predictors that indirectly influence the effectiveness of these educational initiatives. Nonetheless, conflicting evidence exists in this regard, warranting further investigation through additional review studies to draw conclusive insights into the interplay between these predictor factors and the efficacy of educational programs in improving sanitation workers' hygiene knowledge and practices.

Considering these findings, it is recommended that hygiene-related training programs be implemented actively and intensively, presented in a manner that is straightforward and tailored to the educational level of sanitation workers. By aligning the delivery of educational content with the educational background of the workforce, greater engagement and comprehension can be achieved, thereby maximizing the effectiveness of such initiatives.

In conclusion, the evidence supports the notion that educational programs focused on hygiene serve as a vital tool in bolstering the knowledge and practices of sanitation workers. By doing so, these programs contribute to the prevention of hygiene-related infections, including cholera, diarrhoea, hepatitis B and various other diseases, thus fostering a healthier environment for all.

### 5.1. Study Strengths and Limitations

The findings from the reviewed studies underscore that the effectiveness of intervention programs for sanitation workers is not only solely contingent upon the type of intervention employed but is also indirectly influenced by demographic factors such as age, work experience and educational attainment. These factors are pivotal considerations when designing and implementing educational programs aimed at enhancing hygiene knowledge and practices among sanitation workers. By recognizing and accounting for these predictors, interventions can be tailored more effectively to address the specific needs and circumstances of sanitation workers, thereby maximising their impact.

From a practical standpoint, our systematic review serves as a valuable resource for policymakers and stakeholders involved in the implementation of educational hygiene training programs for sanitation workers. By synthesising and analysing existing evidence, this study provides insights that can inform the development of targeted interventions aimed at improving hygiene-related knowledge and practices within this workforce. Moreover, the evidence presented can serve as a foundation for identifying key factors that contribute to the efficacy of such programs, enabling stakeholders to design more nuanced and effective strategies for intervention planning and implementation.

However, it is important to note that the studies included in this systematic review are not representative of all regions globally. The geographical scope of the studies included is limited, with a predominant focus on countries in Africa, Asia and the Middle East. Therefore, there is a potential bias in the included studies due to the geographical focus. In addition, variations in the terminology used to describe sanitation workers across different regions, languages and cultures may impact the generalisability of the findings. Despite employing rigorous search techniques, the potential for variability in terminology highlights the need for caution when interpreting and applying the findings of this review in diverse contexts. Furthermore, there were linguistic limitations placed on the articles that were included, namely, those written in English. Articles published in non-English languages were explicitly omitted since they may introduce prejudice. Due to some of the reviewed studies not providing standard deviation values in their studies, it became quite impossible to perform a meta-analysis on the pre–post mean values.

### 5.2. Study Recommendations

In light of the review findings, the following recommendations are for the Department of Education, policymakers and for further research studies.

#### 5.2.1. Department of Education

The introduction of a curriculum that sufficiently covers hygiene-related diseases in prematric education is highly recommended.

#### 5.2.2. Policymakers

To enhance hygiene knowledge and practices among sanitation workers, policymakers should develop comprehensive guidelines. These guidelines should advocate for the active and intensive implementation of hygiene-related training programs. The training should be delivered in a clear, precise manner and tailored to the educational levels of the sanitation workers to ensure effectiveness and understanding.

#### 5.2.3. Further Research Studies

Additional research is needed to provide definitive insights into how demographic factors influence the effectiveness of educational programmes in enhancing the hygiene knowledge and practices of sanitation workers. Further research is warranted to investigate the effectiveness of behavioural modification strategies in promoting and sustaining positive hygiene practices.

## Figures and Tables

**Table 1:** Summary of studies.

Author	Study design	Location	Aim	Category of sanitation workers	Educational level acquired by the majority of sanitation workers	Type of institution the study participants were affiliated	Intervention method used	Period gap between pre- and postintervention	Outcome	Conclusion
1. Temesgen et al. [25]	Cross-sectional study	Ethiopia	To determine the prevalence of occupational injuries and associated factors among municipal solid waste collectors.	Municipal solid waste workers (MSWW).	Illiterate (56%).	Municipality.	NR.	NR.	- Training in hygiene reduced the likelihood of occupational hygiene injuries by 90% for MSWW. - An average of 28% also lacked adequate hygiene.	- Hygienic training and instruction enhance sanitation workers' hygienic practices.

2. Melaku and Tiruneh [20]	Cross-sectional study	Ethiopia	To investigate occupational health condition and associated factors among municipal solid waste collectors.	Municipal solid waste workers.	Primary education (grades 1–6) (46%).	Municipality.	NR.	NR.	- Only 18.9% of MSWW received educational programme. - Leading to only 10% of the participants using good hygiene practices.	- Due to limited educational training programmes on hygiene received by MSWW, they were widely exposed to hygiene-related diseases.

3. Basavaraj et al. [28]	Observational cross-sectional study.	Bangalore	To assess hygiene knowledge, attitude and practices in biomedical waste management among healthcare workers.	Healthcare sanitary workers.	NR.	Healthcare facility.	NR.	NR.	- Only 15% of the participants received hygiene-related training. - None of the participants were using personal protective equipment (PPE) when handling waste.	- The study concluded that healthcare sanitary workers had poor hygiene knowledge.

4. Kumar et al. [29]	Quasiexperimental study, with the control group.	Pakistan	To evaluate the effectiveness of training intervention to improve the knowledge, attitude and practices of hospital workers on healthcare waste management.	Healthcare sanitary workers.	NR.	Healthcare facility.	Classroom learning (using waste management guidelines and procedures). Practical training method (using practical demonstration).	3 months	- Average mean score for baseline hygiene knowledge was 8.3 and 12.2 average mean score for the post-test. - The hygiene practice was statistically significantly better at post-test (mean = 9.2) opposed to baseline (mean = 2.5).	- The hygiene knowledge and practices of sanitary workers had significantly improved after the implementation of the training.

5. Sapkota et al. [30]	Pre–post-test intervention study.	Nepal	Impact of educational training on hygiene practices among healthcare waste handler.	Healthcare sanitary workers.	NR.	Healthcare facility.	Classroom learning (WHO standards and waste management policies). Handout of printed materials (brochure and posters).	NR.	- Pretest hygiene practice score was 26% (poor), and post-test evaluation score was 86% (excellent).	- The preintervention evaluation score among hospital waste handlers was statistically significantly lower (26%) compared with postintervention score (86%) of waste handlers hygiene practices. - It is evident that poor hygiene practices when handling waste can lead to more than 30 pathogens, including hepatitis B & C, and *E. coli.*

6. Tabash et al. [31]	Pre–post-test intervention study	Palestine	To determine the impact of an educational program regarding pharmaceutical waste management on the knowledge, attitude and practices of healthcare workers.	Healthcare sanitary workers.	Not clear.	Healthcare facility.	Handout of printed materials (posters). E-learning (PowerPoint presentation). Classroom learning (focus group discussion).	6 months	- Mean practice score before the intervention phase was 37% and 78% post the intervention phase. - Mean knowledge score of 41% was identified before the intervention phase and increased to 77% in the postintervention phase.	- The hygiene knowledge and practices of waste collectors was statistically better after the implementation of the training programme.

7. Hamajima et al. [24]	Cross-sectional study.	Bangladesh	To assess the knowledge and practice of healthcare providers regarding medical waste management and to identify any possible barriers related to it.	Healthcare sanitary workers.	Not clear.	Healthcare facility.	NR.	NR.	- More than 76% had inadequate knowledge regarding the hygiene when managing waste, and over 56% of the participants showed poor hygiene practices.	- Low educational level acquired by the participants and the lack of educational training on safe waste management was regarded as the contributing factor of inadequate hygiene knowledge and practice among hospital sanitation workers, thus exposing them to occupational hygiene-related diseases.

8. Onoh et al. [32]	Cross-sectional study	Nigeria	To assess the hygiene knowledge and practices of healthcare waste management among hospital cleaning staff.	Healthcare sanitary workers.	Secondary education (grades 8–12) (56%).	Healthcare facility.	NR.	NR.	- Only 57% of the healthcare sanitary workers who have received educational training on good hygiene practices when handling waste at the entry level. - Only 17% of them had a tertiary level of education. - The mean score of good hygiene practices of 53% was recorded in the study.	- The hygiene knowledge was statistically significantly better among the participants who acquired tertiary education.

9. Millanzi et al. [33]	Descriptive cross-sectional study	Tanzania	To evaluate sanitary workers' KAP about healthcare waste treatment in Dodoma region.	Healthcare sanitary workers.	Primary school (55%).	Healthcare facility.	NR.	NR.	- More than 9% of sanitation workers had never received any kind of formal education. - The majority of the participants (74%) lacked adequate knowledge regarding hygiene. - At least 64% of sanitary staff had poor hygiene practices when discharging their duties.	- The study concluded that different educational background had a significant impact on participants' perception and knowledge about hygiene. - There is a lack of training programme that involve and empower sanitation workers with knowledge and good hygiene practices for handling waste.

10. AbouZeid e al. [34]	Quasiexperimental study design	Egypt	To investigate effectiveness of educational program on utilization of PPE among municipal waste workers at Minia city.	Street sweepers and garbage collectors.	Illiterate (68%).	Municipality.	Classroom learning (group discussion). E-learning (presentations and teaching videos) Handout of printed material (flyers).	1 month	- More than 69% of the participants were illiterate, and over 90% never received any training regarding hygiene. - During the pretest, 41% of the participants were not using PPE when handling waste, and post an educational training, 84% of the waste workers had good hygiene practices. - Over 46% of the participants had poor knowledge about the importance of using PPE, and at the postintervention phase, there was a significant improvement on their hygiene knowledge with the mean score of 82%.	- Lack of hygiene-related training and acquired education level were the most common contributing factors to poor hygiene knowledge and practice among municipal waste workers in the study area.

11. Ben Jmaa et al. [35]	Quasiexperimental study design.	Southern Tunisia	To assess the impact of a training program on knowledge and practical skills of healthcare professionals (HCPs) regarding healthcare waste(HCW) management in a teaching hospital in Southern Tunisia	Healthcare sanitary workers.	Not clear.	Healthcare facility.	Classroom learning (group discussion and lectures). E-learning methods (PowerPoint presentation and videos) Handout of printed material (posters and flyers).	1 month.	- The hygiene knowledge of sanitary staff significantly improved, raising from 54% at baseline to 76% post an educational programme. - The average mean score of hygiene practices significantly improved, raising from 47% pre an educational training to 74% post an educational training programme.	- The implemented educational programme led to a statistically significant improvement in the knowledge and practice of sanitary staff regarding safe waste handling. - Age, educational level acquired and work experience were noted to be independently associated with increasing knowledge change.

12. Mohamed and Mohamed [36]	Quasiexperimental study design.	Egypt	Evaluate the effect of the educational program on occupational health and safety behaviours among sewage workers.	Sewage workers.	Secondary education (44%).	Municipality.	Classroom learning method (teaching in class).	3 months	- Post educational programme, up to 57% of sewage workers had good hygiene knowledge, up from 14% pretest. - Less than 9% of sewage workers had good hygiene practices pretest, while 70% improved at the posteducational intervention.	- In the study, it was evident that there is a positive association between satisfactory hygiene knowledge and age, work experience and acquired educational level.

13. Elnour et al. [37]	Quasiexperimental study design.	Sudan	To assess nursing and sanitation staff knowledge and practice regarding healthcare waste (HCW) management before and after the implementation of an educational intervention program at the main hospitals of the White Nile State in Sudan.	Healthcare sanitary workers.	NR.	Healthcare facility.	Classroom learning (focus group discussion). E-learning (PowerPoint presentation and videos).	Immediately and 1 month later.	- Over 63% never attended any hygiene-related training programme. - The pretest control group showed that only 17% (17/100) of sanitation workers had adequate hygiene knowledge, and improved by up to 59% post-test. - During the pretest phase, 19% of the participants revealed that they never wore PPE when handling waste; however, the post-test findings showed a higher percentage of over 55%.	- The intervention programme had a good effect on sanitation worker's hygiene knowledge and practices.

14. Awad et al. [38]	Quasiexperimental study design.	Egypt	Evaluate the effect of an occupational health program on waste collection workers' knowledge and practices.	Garbage collectors.	Low literacy level (can only read and write) (52%).	Municipality	E-learning (presentation) Handout of printed material (posters and flyers). Practical training method (demonstration).	2 months	- The garbage collectors had a low literacy level. - The pretest mean knowledge score of the participants was 37%, and it significantly improved to a knowledge mean score of 70% for the post-test. - The mean hygiene practice score before the implementation of the educational programme was 31%, and it significantly improved after the programme to 56%.	- After implementing the hygiene-related program, there was a statistically significant difference in the participants' knowledge and safe practices at the post-test compared with their pretest level.

15. Kumar et al. [39]	Quasiexperimental study design	Pakistan	To assess the effectiveness of intensive healthcare waste management (IHWM) training model at two tertiary care hospitals of Rawalpindi city, Pakistan.	Healthcare sanitary workers.	Not clear.	Healthcare facility.	Practical training method (physical demonstration). Classroom learning (WHO guidelines and reminder services).	3 months	- The sanitary staff had a knowledge score of 41% for the baseline study, significantly improved to 65% post an educational training.	- Poor hygiene practices can lead to the infection of waste handlers with hepatitis B and C, cholera and TB. - Age, gender and educational level had no significant association with the hygiene knowledge and practice of the participants. - The educational training had a positive impact on sanitary workers' hygiene knowledge and practice.

Abbreviation: NR, not reported.

**Table 2:** The period gap between the implementation of an educational program and the post test.

Author (year of study)	Period gap between implementation of intervention and the baseline assessment	Period gap between intervention phase and the post-test assessment
AbouZeid et al. (2022) [34]	2 months	1 month
Ben Jmaa et al. (2023) [35]	NR	1 month
Kumar et al. (2016) [29]	Immediately	3 months
Mohamed and Mohamed (2023) [36].	NR	3 months
Elnour et al. (2015) [37]	NR	3 months
Kumar et al. (2015) [39]	Immediately	3 months
Tabash et al. (2016) [31]	Immediately	6 months
Awad et al. (2023) [38]	NR.	2 months
Sapkota et al. (2014) [30]	NR	8 months

Abbreviation: NR, not reported.

**Table 3:** Pre–post intervention hygiene-related knowledge and practice mean and standard deviation scores.

Knowledge	Pre			Post			Post–pre
	Score (%)	*n*	SD	Score (%)	*n*	SD	Difference (%)
Kumar et al. [29].	13	26	3.1	19	26	3	6
Tabash et al. [31].	41	69	21	77	69	11	36
AbouZeid et al. [34].	54	168	2.9	82	168	1.97	28
Ben Jmaa et al. [35]	54	14	—	76	14	—	22
Mohamed and Mohamed [36].	14	230	4.01	57	230	0.45	43
Elnour et al. [37].	17	100	—	59	100	—	42
Awad et al. [38]	37	95	—	70	95	—	33
Kumar et al. [39].	7.59	27	2.45	8.3	26	3.12	0.71

**Practice**	**Pre**			**Post**			**Post**–**pre**
	**Score (%)**	***n***	**SD**	**Score (%)**	***n***	**SD**	**Difference (%)**

Kumar et al. [29].	11	26	3.8	15	26	3	4
Sapkota et al. [30].	26	40	—	86	40	—	60
Tabash et al. [31].	37	69	26	78	69	30	41
AbouZeid et al. [34].	41	168	1.79	84	168	1.44	43
Ben Jmaa et al. [35]	47	14	—	74	14	—	27
Mohamed and Mohamed [36].	9	230	4.49	70	230	0.41	61
Elnour et al. [37].	42	100	—	55	100	—	13
Awad et al. [38]	31	95	—	56	95	—	25

*Note:* —: not provided in the publication.

## Data Availability

The data that support the findings of this study are available from the corresponding author upon reasonable request.

## Supporting Information

Supporting Information 1 Annexure S1: Search strategy.

Supporting Information 2 Table S2: Screened studies for full text (included and excluded studies in the review).

Supporting Information 3 Checklist S3: JBI Critical Appraisal Checklist of individual studies.

Supporting Information 4 Table S4: PRISMA checklist for abstracts.

Supporting Information 5 Table S5: PRISMA checklist for systematic review.
