# Supplementary material for: Impact of Educational Intervention on Hygiene Knowledge and Practices of Sanitation Workers Globally: A Systematic Review
Source: Scientifica (Cairo). 2025 Aug 25;2025:3265559. doi: 10.1155/sci5/3265559 (PMC12401612; doi:10.1155/sci5/3265559)
Supplement: Supporting Information 1 — Annexure S1: Search strategy. [file 3265559.f1.docx]

**IMPACT OF EDUCATIONAL PROGRAMS ON SANITATION WORKERS’ HYGIENE-RELATED, KNOWLEDGE AND PRACTICES: A SYSTEMATIC REVIEW**

**Population** - Sanitation workers

**Intervention** - Educational programmes

**Comparison** – Hygiene knowledge and practices, and the spread of infectious diseases.

**Outcome** – Impact educational programs have on sanitation workers’ hygiene knowledge and practices, and in the spread of infectious diseases in the communities.

**Search terms**

- Sanitation workers, waste collectors, garbage collectors.
- Educational programs, Training program
- Hygiene knowledge and practices

Exclusion and inclusion

Exclusion:

- The studies not focussing on the hygiene knowledge and practices, and educational programs among sanitation workers.
- Publication of duplicated studies, and
- Grey literature will be excluded in the review.

Inclusion:

- Studies will be eligible for inclusion if they were published in a peer reviewed journal, written/translated into English in the past 10 years (2013-2023).
- Quantitative studies reported on educational programs given to sanitation workers’ hygiene-related knowledge, and practices

**EBSCOHost:**

**PubMed: History and Search Details**

| **Search** | **Actions** | **Details** | **Query** | **Results** | **Time** |
| --- | --- | --- | --- | --- | --- |
| #10 |  |  | Search: **(Sanitation workers OR waste collectors OR Garbage collectors) AND (Educational programs OR Training programs) AND (Hygiene knowledge and practices)** Filters: **Free full text, Full text, English, from 2013/1/1 - 2023/12/30** | [32](https://pubmed.ncbi.nlm.nih.gov/?term=%28Sanitation+workers+OR+waste+collectors+OR+Garbage+collectors%29+AND+%28Educational+programs+OR+Training+programs%29+AND+%28Hygiene+knowledge+and+practices%29&filter=simsearch2.ffrft&filter=simsearch3.fft&filter=dates.2013%2F1%2F1-2023%2F12%2F30&filter=lang.english&sort=relevance) | 11:18:52 |
| #9 |  |  | Search: **Hygiene knowledge and practices** Filters: **Free full text, Full text, English, from 2013/1/1 - 2023/12/30** | [3,454](https://pubmed.ncbi.nlm.nih.gov/?term=Hygiene+knowledge+and+practices&sort=&filter=simsearch2.ffrft&filter=simsearch3.fft&filter=dates.2013%2F1%2F1-2023%2F12%2F30&filter=lang.english) | 11:00:27 |
| #8 |  |  | Search: **Training programs OR Educational programs** Filters: **Free full text, Full text, English, from 2013/1/1 - 2023/12/30** | [628,160](https://pubmed.ncbi.nlm.nih.gov/?term=Training+programs+OR+Educational+programs&filter=simsearch2.ffrft&filter=simsearch3.fft&filter=dates.2013%2F1%2F1-2023%2F12%2F30&filter=lang.english&sort=relevance) | 10:58:54 |
| #7 |  |  | Search: **Training programs** Filters: **Free full text, Full text, English, from 2013/1/1 - 2023/12/30** | [608,648](https://pubmed.ncbi.nlm.nih.gov/?term=Training+programs&filter=simsearch2.ffrft&filter=simsearch3.fft&filter=dates.2013%2F1%2F1-2023%2F12%2F30&filter=lang.english&sort=relevance) | 10:58:29 |
| #6 |  |  | Search: **Educational programs** Filters: **Free full text, Full text, English, from 2013/1/1 - 2023/12/30** | [149,674](https://pubmed.ncbi.nlm.nih.gov/?term=Educational+programs&sort=&filter=simsearch2.ffrft&filter=simsearch3.fft&filter=dates.2013%2F1%2F1-2023%2F12%2F30&filter=lang.english) | 10:58:13 |
| #5 |  |  | Search: **Waste collectors OR Garbage collectors OR Sanitation workers** Filters: **Free full text, Full text, English, from 2013/1/1 - 2023/12/30** | [1,269](https://pubmed.ncbi.nlm.nih.gov/?term=Waste+collectors+OR+Garbage+collectors+OR+Sanitation+workers&filter=simsearch2.ffrft&filter=simsearch3.fft&filter=dates.2013%2F1%2F1-2023%2F12%2F30&filter=lang.english&sort=relevance) | 10:57:09 |
| #4 |  |  | Search: **garbage collectors** Filters: **Free full text, Full text, English, from 2013/1/1 - 2023/12/30** | [24](https://pubmed.ncbi.nlm.nih.gov/?term=garbage+collectors&filter=simsearch2.ffrft&filter=simsearch3.fft&filter=dates.2013%2F1%2F1-2023%2F12%2F30&filter=lang.english&sort=relevance) | 10:39:49 |
| #3 |  |  | Search: **Waste collectors** Filters: **Free full text, Full text, English, from 2013/1/1 - 2023/12/30** | [164](https://pubmed.ncbi.nlm.nih.gov/?term=Waste+collectors&sort=&filter=simsearch2.ffrft&filter=simsearch3.fft&filter=dates.2013%2F1%2F1-2023%2F12%2F30&filter=lang.english) | 10:31:51 |
| #2 |  |  | Search: **sanitation workers** Filters: **Free full text, Full text, English, from 2013/1/1 - 2023/12/30** | [1,115](https://pubmed.ncbi.nlm.nih.gov/?term=sanitation+workers&filter=simsearch2.ffrft&filter=simsearch3.fft&filter=dates.2013%2F1%2F1-2023%2F12%2F30&filter=lang.english&sort=relevance) | 10:30:40 |

**Medline: History and Search Details**

| [Search ID#](javascript:__doPostBack('ctl00$ctl00$FindField$FindField$historyControl$ReorderHistoryLink',''))Search TermsSearch OptionsActionsS9 | ( Sanitation workers OR Waste collectors OR Garbage collectors ) AND ( Educational programs OR Training programs ) AND ( Hygiene knowledge and practices ) | **Limiters** - Peer Reviewed; Publication Date: 20130101-20231231; Citation Subset: MEDLINE; Publication Type: Journal Article; Language: English | **Expanders** - Apply related words; Apply equivalent subjects |
| --- | --- | --- | --- |
| **Search modes** - Find all my search terms | [**View Results**](javascript:__doPostBack('ctl00$ctl00$FindField$FindField$historyControl$HistoryRepeater$ctl00$linkResults','')) (5) | [**View Details**](javascript:showShDetails(%22ctl00_ctl00_FindField_FindField_historyControl_ctrlPopup%22,%20%22S9%22,%20true);) | [**Edit**](https://0-web.p.ebscohost.com.wam.seals.ac.za/Legacy/Views/UserControls/EHOST/) |
| S8 | Hygiene knowledge and practices | **Limiters** - Peer Reviewed; Publication Date: 20130101-20231231; Citation Subset: MEDLINE; Publication Type: Journal Article; Language: English | **Expanders** - Apply related words; Apply equivalent subjects |
| **Search modes** - Find all my search terms | [**View Results**](javascript:__doPostBack('ctl00$ctl00$FindField$FindField$historyControl$HistoryRepeater$ctl01$linkResults','')) (3,625) | [**View Details**](javascript:showShDetails(%22ctl00_ctl00_FindField_FindField_historyControl_ctrlPopup%22,%20%22S8%22,%20true);) | [**Edit**](https://0-web.p.ebscohost.com.wam.seals.ac.za/Legacy/Views/UserControls/EHOST/) |
| S7 | Educational programs OR Training programs | **Limiters** - Peer Reviewed; Publication Date: 20130101-20231231; Citation Subset: MEDLINE; Publication Type: Journal Article; Language: English | **Expanders** - Apply related words; Apply equivalent subjects |
| **Search modes** - Find all my search terms | [**View Results**](javascript:__doPostBack('ctl00$ctl00$FindField$FindField$historyControl$HistoryRepeater$ctl02$linkResults','')) (91,350) | [**View Details**](javascript:showShDetails(%22ctl00_ctl00_FindField_FindField_historyControl_ctrlPopup%22,%20%22S7%22,%20true);) | [**Edit**](https://0-web.p.ebscohost.com.wam.seals.ac.za/Legacy/Views/UserControls/EHOST/) |
| S6 | Training programs | **Limiters** - Peer Reviewed; Publication Date: 20130101-20231231; Citation Subset: MEDLINE; Publication Type: Journal Article; Language: English | **Expanders** - Apply related words; Apply equivalent subjects |
| **Search modes** - Find all my search terms | [**View Results**](javascript:__doPostBack('ctl00$ctl00$FindField$FindField$historyControl$HistoryRepeater$ctl03$linkResults','')) (69,821) | [**View Details**](javascript:showShDetails(%22ctl00_ctl00_FindField_FindField_historyControl_ctrlPopup%22,%20%22S6%22,%20true);) | [**Edit**](https://0-web.p.ebscohost.com.wam.seals.ac.za/Legacy/Views/UserControls/EHOST/) |
| S5 | Educational programs | **Limiters** - Peer Reviewed; Publication Date: 20130101-20231231; Citation Subset: MEDLINE; Publication Type: Journal Article; Language: English | **Expanders** - Apply related words; Apply equivalent subjects |
| **Search modes** - Find all my search terms | [**View Results**](javascript:__doPostBack('ctl00$ctl00$FindField$FindField$historyControl$HistoryRepeater$ctl04$linkResults','')) (30,661) | [**View Details**](javascript:showShDetails(%22ctl00_ctl00_FindField_FindField_historyControl_ctrlPopup%22,%20%22S5%22,%20true);) | [**Edit**](https://0-web.p.ebscohost.com.wam.seals.ac.za/Legacy/Views/UserControls/EHOST/) |
| S4 | Sanitation workers OR Waste collectors OR Garbage collectors | **Limiters** - Peer Reviewed; Publication Date: 20130101-20231231; Citation Subset: MEDLINE; Publication Type: Journal Article; Language: English | **Expanders** - Apply related words; Apply equivalent subjects |
| **Search modes** - Find all my search terms | [**View Results**](javascript:__doPostBack('ctl00$ctl00$FindField$FindField$historyControl$HistoryRepeater$ctl05$linkResults','')) (734) | [**View Details**](javascript:showShDetails(%22ctl00_ctl00_FindField_FindField_historyControl_ctrlPopup%22,%20%22S4%22,%20true);) | [**Edit**](https://0-web.p.ebscohost.com.wam.seals.ac.za/Legacy/Views/UserControls/EHOST/) |
| S3 | Garbage collectors | **Limiters** - Peer Reviewed; Publication Date: 20130101-20231231; Citation Subset: MEDLINE; Publication Type: Journal Article; Language: English | **Expanders** - Apply related words; Apply equivalent subjects |
| **Search modes** - Find all my search terms | [**View Results**](javascript:__doPostBack('ctl00$ctl00$FindField$FindField$historyControl$HistoryRepeater$ctl06$linkResults','')) (20) | [**View Details**](javascript:showShDetails(%22ctl00_ctl00_FindField_FindField_historyControl_ctrlPopup%22,%20%22S3%22,%20true);) | [**Edit**](https://0-web.p.ebscohost.com.wam.seals.ac.za/Legacy/Views/UserControls/EHOST/) |
| S2 | Waste collectors | **Limiters** - Peer Reviewed; Publication Date: 20130101-20231231; Citation Subset: MEDLINE; Publication Type: Journal Article; Language: English | **Expanders** - Apply related words; Apply equivalent subjects |
| **Search modes** - Find all my search terms | [**View Results**](javascript:__doPostBack('ctl00$ctl00$FindField$FindField$historyControl$HistoryRepeater$ctl07$linkResults','')) (315) | [**View Details**](javascript:showShDetails(%22ctl00_ctl00_FindField_FindField_historyControl_ctrlPopup%22,%20%22S2%22,%20true);) | [**Edit**](https://0-web.p.ebscohost.com.wam.seals.ac.za/Legacy/Views/UserControls/EHOST/) |
| S1 | Sanitation workers | **Limiters** - Peer Reviewed; Publication Date: 20130101-20231231; Citation Subset: MEDLINE; Publication Type: Journal Article; Language: English | **Expanders** - Apply related words; Apply equivalent subjects |
| **Search modes** - Find all my search terms | [**View Results**](javascript:__doPostBack('ctl00$ctl00$FindField$FindField$historyControl$HistoryRepeater$ctl08$linkResults','')) (419) | [**View Details**](javascript:showShDetails(%22ctl00_ctl00_FindField_FindField_historyControl_ctrlPopup%22,%20%22S1%22,%20true);) | [**Edit**](https://0-web.p.ebscohost.com.wam.seals.ac.za/Legacy/Views/UserControls/EHOST/) |

**Google Scholar: History and Search Details**

**
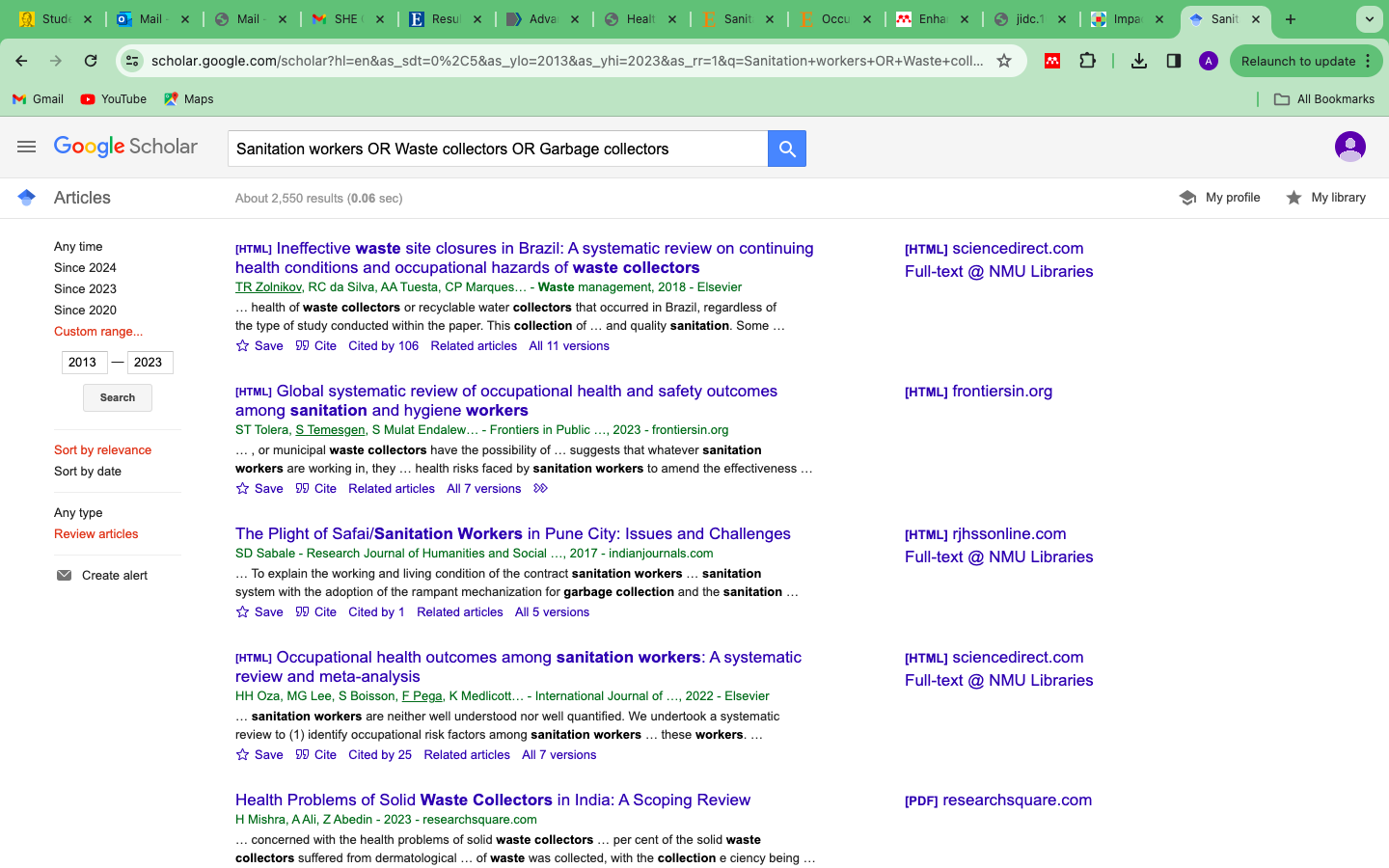
**

**
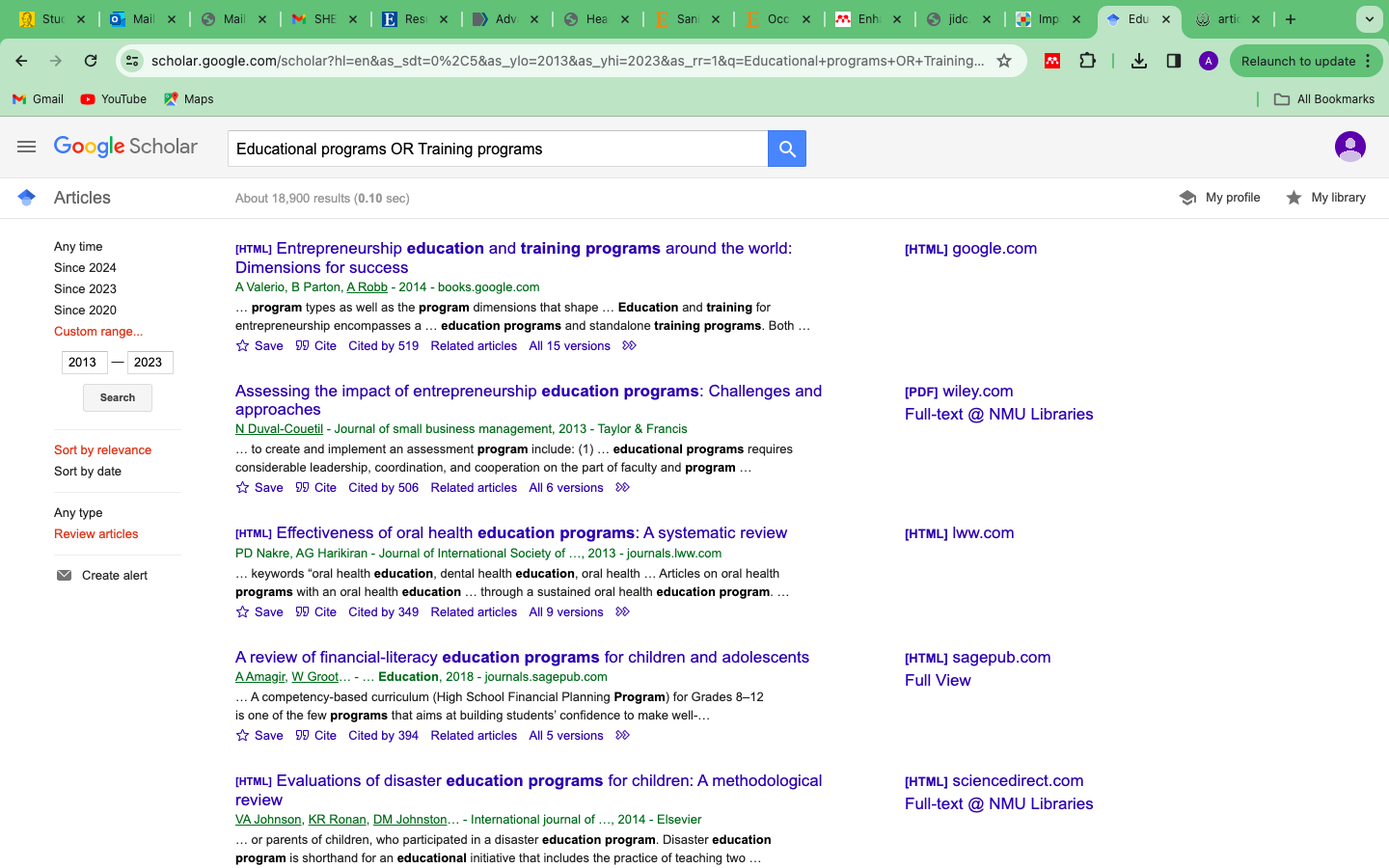
**

**
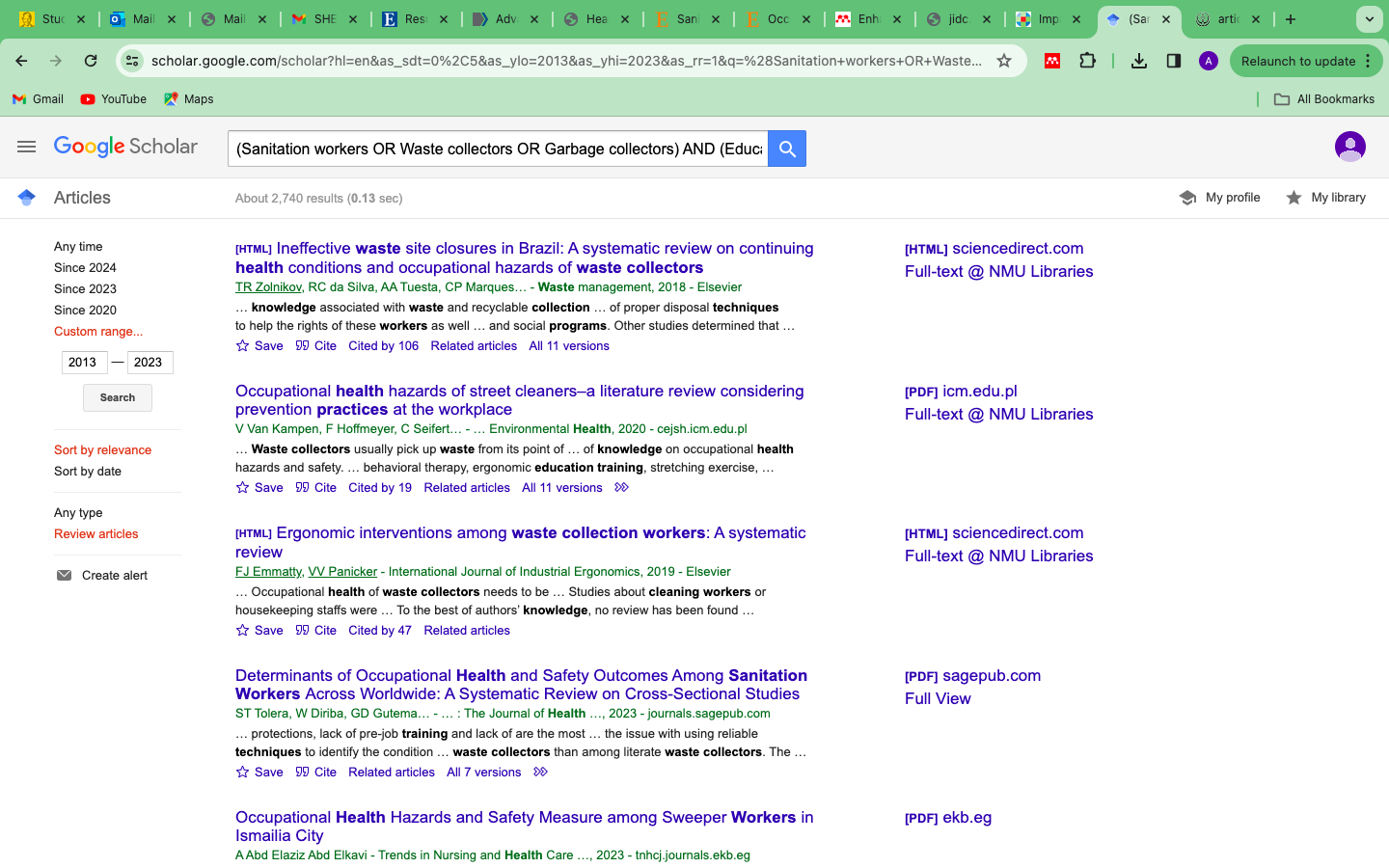
**

In addition to the aforementioned technique, the following key question was utilized to search for study-related articles in this database.

- Educational impact on waste collectors’ hygiene knowledge and practices.

**Documents identified through data searching Boolean operators “AND & OR”= (N=2 777)**

- Medline = 5
- PubMed = 32
- Google scholar = 2 740

**Duplicated studies during the search from all three databases (N = 2 744)**

- Duplicate = 33

Total of 2 744 articles were retained after the duplicated studies (33) were removed, out of those 2 671 were also removed due to the following reasons:

- Title and abstract were not aligning to the objectives
- The articles were not peer reviewed.
- The articles did not meet the inclusion criteria for the review.

**Screened for full text review. (N = 73)**

- 73 studies were selected for full text review

Eligible studies included in the review (N= 15)

- 15 studies were eligible for inclusion in the review.
