# Supplementary material for: Impact of Educational Intervention on Hygiene Knowledge and Practices of Sanitation Workers Globally: A Systematic Review
Source: Scientifica (Cairo). 2025 Aug 25;2025:3265559. doi: 10.1155/sci5/3265559 (PMC12401612; doi:10.1155/sci5/3265559)
Supplement: Supporting Information 2 — Table S2: Screened studies for full text (included and excluded studies in the review). [file 3265559.f2.docx]

**Studies screened full text (included & excluded)**

| No. | Author/Year of study | Study title | Study aim/s | Study location | Summary of findings | Included | Excluded | Reason for exclusion |
| --- | --- | --- | --- | --- | --- | --- | --- | --- |
| 1 | Temesgen et al., 2022). | Occupational injuries and associated factors among municipal solid waste collectors in Harar Town, Eastern Ethiopia: A cross sectional study. | To determine the prevalence of occupational injuries and associated factors among municipal solid waste collectors | Ethiopia | Health and safety training and educational level acquired have a significant impact on good hygiene practice and on occupational injuries. MSWW who received training had 90% lower risk of getting occupational hygiene related injuries. 60.4% Exposed to occupational hygiene injuries. Average of 28% were not practicing good hygiene practice. | Yes |  |  |
| 2 | (Melaku & Tiruneh, 2020). | Occupational health conditions and associated factors among municipal solid waste collectors in Addis Ababa, Ethiopia. | To investigate occupational health condition and associated factors among municipal solid waste collectors. | Ethiopia | Due to lack educational training on hygiene and poor hygiene practices MSWW were widely exposed to respiratory infections (45%) and skin symptom (34%). 18.9% of MSWW received educational program. Leading to at least 10% of the participant used gloves when handling waste, and 73% never attended to their personal hygiene after work. | Yes |  |  |
| 3 | (Basavaraj et al., 2021). | To assess the knowledge, attitude and practices in biomedical waste management among health care workers in dedicated COVID hospital in Bangalore. | To assess hygiene knowledge, attitude and practices in biomedical waste management among health care workers. | Bangalore | The study concluded that Healthcare housekeeping staff had poor hygiene knowledge compared to nurses and doctors. This was due, to the lack of educational training among housekeeping workers. The study further highlights the significance of implementing educational programs, as a tool of enhancing hospital Housekeepers’ hygiene knowledge and practices. | Yes |  |  |
| 4 | (Kumar et al., 2016). | Impact of waste management training intervention on knowledge, attitude and practices of teaching hospital workers in Pakistan. | To evaluate the effectiveness of training intervention to improve the knowledge, attitude and practices of hospital workers on health care waste management. | Pakistan | The hygiene knowledge and practices of sanitary workers had significantly improved after the implementation of the training. The hygiene training when combined with other innovative approaches has been proven to be one of the most effective strategy of improving hygiene knowledge and practices. | Yes |  |  |
| 5 | (Eskezi et al., 2016) | Prevalence and associated factors of occupational injuries among municipal solid waste collectors in four zones of Amhara region, Northwest Ethiopia. | To assess the prevalence of occupational injuries and its associated factors among municipal solid waste collectors in Ethiopia. | Ethiopia | The prevalence of occupational injuries was found to be less prominent compared to previous studies conducted in the same study area. The following interventions were expected to cope with the problem: job rotation, improvement of employees’ wage, and guidelines related to maximum production limits. |  | Yes | The study did not provide pertinent information regarding the impact of hygiene-related intervention on participants’ knowledge and practices. |
| 6 | Sapkota et al., 2014) | Impact of intervention on healthcare waste management practices in a tertiary care governmental hospital of Nepal. | To determine the Impact of educational training on hygiene practices among healthcare waste handler. | Nepal | The pre-intervention evaluation score among hospital waste handlers was statistically significantly lower (26%) compared to post-intervention score (86%) of waste handlers hygiene practices.  It also evident that poor hygiene practices when handling waste can lead to more than 30 pathogens, including Hepatitis B & C, E. *coli.* | Yes |  |  |
| 7 | (Fatmi et al., 2022) | Health related quality of life amongst Sewerage and Sanitation Workers of Karachi, Pakistan. | To assess the impact of occupational and socio-demographic factors on the health related quality of life among Sewage and Sanitation workers in Karachi. | Pakistan | It was observed that the standard of living of sanitation workers is below par. This is due to insufficient income to meet their basic needs, and due to poor practices when handling waste. |  | Yes | This study was mainly focussing on the quality of life sanitation workers are experiencing and associated factors. Therefore, this study did not assess the impact of educational programs regarding hygiene on sanitation workers’ knowledge and practices. |
| 8 | (Tabash et al., 2016). | Impact of an intervention programme on knowledge, attitude and practice of healthcare staff regarding pharmaceutical waste management, Gaza, Palestine. | To determine the impact of an educational program regarding pharmaceutical waste management on the knowledge, attitude, and practices of healthcare workers | Palestine | Educational intervention should be used as an effective tool of protecting human health and the environment. The hygiene knowledge and practices of waste collectors was statistically better after the implementation of the training program. | Yes |  |  |
| 9 | (Sarker et al., 2014) | Evaluation of knowledge, practices, and possible barriers among healthcare providers regarding medical waste management in Dhaka, Bangladesh. | To assess the knowledge and practice of healthcare providers regarding medical waste management, and to identify any possible barriers related to it. | Bangladesh | Inadequate knowledge and practice among the cleaning staff, was due to the lack of basic education acquired by this group of participants. Lack of educational training on safe waste management was regarded as the contributing factor of inadequate hygiene knowledge and practice among hospital cleaning staff, thus exposing them to occupational hygiene-related diseases. Poor hygiene knowledge and practice among the cleaning staff when handling hospital waste was evident. Only 57% of cleaning staff who ever received training, and 76% had inadequate knowledge regarding the hygiene when managing waste, and over 56% of the participants showed poor hygiene practices. | Yes |  |  |
| 10 | (Onoh et al., 2019). | Knowledge and practices of health-care waste management among health workers in Lassa fever treatment facility in Southeast Nigeria. | To assess the hygiene knowledge and practices of healthcare waste management among hospital cleaning staff. | Nigeria | The study concluded that there is a need of educational training on good waste management, thus enhancing their hygiene knowledge and practice and protecting the waste handlers against the transmission of hygiene-related disease when sorting the waste. The hygiene knowledge was statistically significantly better among the participants who acquired tertiary level of education.  Only 57% of the participants who received educational training on good hygiene practices when handling waste at entry level. Only 17% of the participants had tertiary level of education. As a result only 45% of the cleaning staff knew that poor hygiene practices when handling waste can transmit hygiene-related diseases. The mean score of good hygiene practices of 53% was recorded in the study. | Yes |  |  |
| 11 | (Singh et al., 2020) | Effectiveness of a training program about bio-medical waste management on the knowledge and practices of health-care professionals at a tertiary care teaching institute of North India. | To evaluate the impact hygiene-related educational training have on hygiene knowledge and practice of selected health-care professionals at tertiary care teaching institute of North India. | India | A statistical significant improvement in the hygiene knowledge of health-care professionals (nurses and laboratory technicians) following the implementation of educational program was evident. |  | Yes | The study met most of the requirements for inclusion into this review. However, the sanitation workers were not the studied population. |
| 12 | (Millanzi et al., 2023). | Knowledge, attitude, and perceived practice of sanitary workers on healthcare waste management: A descriptive cross-sectional study in Dodoma region, Tanzania. | To evaluate sanitary workers’ Knowledge, Attitude and Practices (KAP) about healthcare waste treatment in Dodoma region. | Tanzania | The majority of sanitary employees did not understand how to handle healthcare waste, despite being the closest person to the collection, transport, and storage of healthcare waste. The study also concluded that different educational background had a significant influence on participants’ perception and knowledge about hygiene.  There is lack of training program that involve and empower sanitation workers with knowledge and good hygiene practice of handling waste.  Over 55% of the participants had only completed their primary school (Education level), and 9% of them had never received any kind of formal education. The majority of the participants (74%) lacked adequate knowledge as regarding hygienic way of handling waste. Over 64% of sanitary staff had poor hygiene practices when discharging their duties. | Yes |  |  |
| 13 | (AbouZeid et al., 2022). | Effect of an educational program on utilization of Personal Protective Equipment among municipal waste workers at Minia city, Egypt. | To investigate effectiveness of educational program on utilization of PPE among municipal waste workers at Minia city. | Egypt | The study revealed that over 60% of waste workers were widely exposed to hygiene-related disease, due to non-compliance with the use of PPE when discharging their duties. Lack of hygiene-related training and acquired education level, were the most common contributing factors to poor hygiene knowledge and practice among municipal waste workers in the study area. | Yes |  |  |
| 14 | (Ben Jmaa et al., 2023) | Effectiveness of a training intervention about healthcare waste management on the knowledge and practical skills of healthcare professionals in a teaching hospital of Southern Tunisia. | To assess the impact of a training program on knowledge and practical skills of healthcare professionals (HCPs) regarding healthcare waste(HCW) management in a Teaching Hospital in Southern Tunisia. | Southern Tunisia | The implemented educational program led to statistically significant improvement in the in the knowledge and practice among sanitary staff regarding safe waste handling. Age, educational level acquired, and work experience were noted to be independently associated with increasing knowledge change.  The hygiene knowledge of sanitary staff significantly improved, rising from 54% at baseline to 76% post an educational program. The average mean score of hygiene practices significantly improved , rising from 47% pre an educational training program to 74% post an educational training program. | Yes |  |  |
| 15 | (Udayanga et al., 2023) | Knowledge, perceptions and practices on healthcare waste management and associated occupational health hazards among healthcare professionals in the Colombo District, Sri Lanka: a cross-sectional study. | To evaluate the hygiene knowledge and practices of healthcare professionals when handling healthcare waste, and to determine the associated risk factors influencing occupational health hazards related to healthcare waste. | Sri Lanka | The majority of the participants showed a satisfactory hygiene knowledge (77%) and practices (54%) regarding healthcare waste management. However, training and awareness training programs are still recommended among healthcare professional. |  | Yes | The sanitation workers were not the studied population. |
| 16 | Mohamed & Mohamed, 2023). | Effect of education program on occupational health and safety behaviors among sewage workers in Egypt. | Evaluate the effect of the educational program on occupational health and safety behaviours among sewage workers. | Egypt | For the pre-test only 14% of sewage workers who had a satisfactory hygiene knowledge, and a significant increase of up to 57% of worker who had a satisfactory hygiene knowledge post an educational training. Only 9% of sewage worker who had a satisfactory hygiene practice for the pre-test, compared to 70% significant improvement on their hygiene practices post an educational intervention. | Yes |  |  |
| 17 | Elnour et al., 2015). | Impact of health education on knowledge and practice of hospital staff with regard to healthcare waste management at White Nile State main hospital, Sudan. | To assess nursing and sanitation staff knowledge and practice regarding Healthcare Waste (HCW) management before and after the implementation of an educational intervention program at the main hospitals of the White Nile State in Sudan. | Sudan | The period gap between the pre-post assessment had no impact on the improvement of participants’ hygiene knowledge and practice. The intervention program had a good effect on sanitation worker’s hygiene knowledge and practice.  The results of the control group (pre-test) shows that only 17% (17/100) of the worker who had satisfactory hygiene knowledge when handling waste. Post-test results (3-months later) showed a significant improvement of up to 59% of sanitation staff who had a satisfactory hygiene knowledge. During the pre-test 19% of the participant revealed that, they never worn PPE when handling waste, and over 63% never attended any hygiene-related training program. Only 42% of the participants had good hygiene practices, compared to the post-test results of more than 55%. | Yes |  |  |
| 18 | (Awad et al., 2023). | An occupational health program for waste collectors workers. | Evaluate the effect of an occupational health program on waste collection workers' knowledge and practices. | Egypt | There was a statistically significant difference in the participants’ knowledge and safe practices at the post-test compared with their pre-test level after implementing the occupational health program.  The garbage collectors had a low literacy level. The study also concluded that poor hygiene knowledge and practice when handling waste can lead to hygiene-related diseases, mainly resulting from hand cuts. | Yes |  |  |
| 19 | Kumar et al., 2015). | Effectiveness of intensive healthcare waste management training model among health professionals at teaching hospitals of Pakistan: a quasi-experimental study. | To assess the effectiveness of Intensive healthcare waste management (IHWM) training model at two tertiary care hospitals of Rawalpindi city, Pakistan. | Pakistan | Poor hygiene practice can lead to the infection of waste handler with Hepatitis B & C, cholera and TB. Age, gender and educational level had no significant association with hygiene knowledge and practice of the participants. The educational training had a positive impact on sanitary workers’ hygiene knowledge and practice. | Yes |  |  |
