# Supplementary material for: Impact of Educational Intervention on Hygiene Knowledge and Practices of Sanitation Workers Globally: A Systematic Review
Source: Scientifica (Cairo). 2025 Aug 25;2025:3265559. doi: 10.1155/sci5/3265559 (PMC12401612; doi:10.1155/sci5/3265559)
Supplement: Supporting Information 3 — Checklist S3: JBI Critical Appraisal Checklist of individual studies. [file 3265559.f3.docx]

checklist for analytical cross sectional studies

Critical Appraisal tools for use in JBI Systematic Reviews

Introduction

JBI is an JBI is an international research organisation based in the Faculty of Health and Medical Sciences at the University of Adelaide, South Australia. JBI develops and delivers unique evidence-based information, software, education and training designed to improve healthcare practice and health outcomes. With over 70 Collaborating Entities, servicing over 90 countries, JBI is a recognised global leader in evidence-based healthcare.

## JBI Systematic Reviews

The core of evidence synthesis is the systematic review of literature of a particular intervention, condition or issue. The systematic review is essentially an analysis of the available literature (that is, evidence) and a judgment of the effectiveness or otherwise of a practice, involving a series of complex steps. JBI takes a particular view on what counts as evidence and the methods utilised to synthesise those different types of evidence. In line with this broader view of evidence, JBI has developed theories, methodologies and rigorous processes for the critical appraisal and synthesis of these diverse forms of evidence in order to aid in clinical decision-making in healthcare. There now exists JBI guidance for conducting reviews of effectiveness research, qualitative research, prevalence/incidence, etiology/risk, economic evaluations, text/opinion, diagnostic test accuracy, mixed-methods, umbrella reviews and scoping reviews. Further information regarding JBI systematic reviews can be found in the [JBI Evidence Synthesis Manual](https://jbi-global-wiki.refined.site/space/MANUAL).

## JBI Critical Appraisal Tools

All systematic reviews incorporate a process of critique or appraisal of the research evidence. The purpose of this appraisal is to assess the methodological quality of a study and to determine the extent to which a study has addressed the possibility of bias in its design, conduct and analysis. All papers selected for inclusion in the systematic review (that is – those that meet the inclusion criteria described in the protocol) need to be subjected to rigorous appraisal by two critical appraisers. The results of this appraisal can then be used to inform synthesis and interpretation of the results of the study. JBI Critical appraisal tools have been developed by the JBI and collaborators and approved by the JBI Scientific Committee following extensive peer review. Although designed for use in systematic reviews, JBI critical appraisal tools can also be used when creating Critically Appraised Topics (CAT), in journal clubs and as an educational tool.

JBI Critical Appraisal Checklist for
analytical cross sectional studies

Reviewer : A. Ntunja Date______01 May 2024_

Author___Kumar et al. (2015)_ Year__2015_______ Record Number___1______

|  | Yes | No | Unclear | Not applicable |
| --- | --- | --- | --- | --- |
| 1. Were the criteria for inclusion in the sample clearly defined? | x | □ | □ | □ |
| 1. Were the study subjects and the setting described in detail? | x | □ | □ | □ |
| 1. Was the exposure measured in a valid and reliable way? | x | □ | □ | □ |
| 1. Were objective, standard criteria used for measurement of the condition? | x | □ | □ | □ |
| 1. Were confounding factors identified? | □ |  | x | □ |
| 1. Were strategies to deal with confounding factors stated? | □ | x | □ | □ |
| 1. Were the outcomes measured in a valid and reliable way? | x | □ | □ | □ |
| 1. Was appropriate statistical analysis used? | x | □ | □ | □ |

Overall appraisal: Include x Exclude □ Seek further info □. 6/8= 75%

Comments (Including reason for exclusion)

________________________________________________________________________________________________________________________________________________________________________________________________________________________________________________________________________________________________

JBI Critical Appraisal Checklist for
analytical cross sectional studies

Reviewer __A. Ntunja_________Date__01 May 2024_________

Author______Elnour et al. (2015)___ Year___2015______ Record Number____2_____

|  | Yes | No | Unclear | Not applicable |
| --- | --- | --- | --- | --- |
| 1. Were the criteria for inclusion in the sample clearly defined? | x | □ | □ | □ |
| 1. Were the study subjects and the setting described in detail? | x | □ | □ | □ |
| 1. Was the exposure measured in a valid and reliable way? | x | □ | □ | □ |
| 1. Were objective, standard criteria used for measurement of the condition? | x | □ | □ | □ |
| 1. Were confounding factors identified? | □ | x | □ | □ |
| 1. Were strategies to deal with confounding factors stated? | □ | x | □ | □ |
| 1. Were the outcomes measured in a valid and reliable way? | x | □ | □ | □ |
| 1. Was appropriate statistical analysis used? | x | □ | □ | □ |

Overall appraisal: Include x Exclude □ Seek further info □. 6/8= 75%

Comments (Including reason for exclusion)

________________________________________________________________________________________________________________________________________________________________________________________________________________________________________________________________________________________________

JBI Critical Appraisal Checklist for
analytical cross sectional studies

Reviewer __A. Ntunja_________Date__01 May 2024_________

Author___Awad et al. (2023)______ Year___2023______ Record Number__3______

|  | Yes | No | Unclear | Not applicable |
| --- | --- | --- | --- | --- |
| 1. Were the criteria for inclusion in the sample clearly defined? | x | □ | □ | □ |
| 1. Were the study subjects and the setting described in detail? | x | □ | □ | □ |
| 1. Was the exposure measured in a valid and reliable way? | x | □ | □ | □ |
| 1. Were objective, standard criteria used for measurement of the condition? | x | □ | □ | □ |
| 1. Were confounding factors identified? | x |  | □ | □ |
| 1. Were strategies to deal with confounding factors stated? | x |  | □ | □ |
| 1. Were the outcomes measured in a valid and reliable way? | x | □ | □ | □ |
| 1. Was appropriate statistical analysis used? | x | □ | □ | □ |

Overall appraisal: Include x Exclude □ Seek further info □. 8/8=100%

Comments (Including reason for exclusion)

________________________________________________________________________________________________________________________________________________________________________________________________________________________________________________________________________________________________

JBI Critical Appraisal Checklist for
analytical cross sectional studies

Reviewer __A. Ntunja_________Date__01 May 2024_________

Author_____ Mohamed & Mohamed, 2023____ Year___2023______ Record Number__4______

|  | Yes | No | Unclear | Not applicable |
| --- | --- | --- | --- | --- |
| 1. Were the criteria for inclusion in the sample clearly defined? | x | □ | □ | □ |
| 1. Were the study subjects and the setting described in detail? | x | □ | □ | □ |
| 1. Was the exposure measured in a valid and reliable way? | x | □ | □ | □ |
| 1. Were objective, standard criteria used for measurement of the condition? | x | □ | □ | □ |
| 1. Were confounding factors identified? | x |  | □ | □ |
| 1. Were strategies to deal with confounding factors stated? | x |  | □ | □ |
| 1. Were the outcomes measured in a valid and reliable way? | x | □ | □ | □ |
| 1. Was appropriate statistical analysis used? | x | □ | □ | □ |

Overall appraisal: Include x Exclude □ Seek further info □. 8/8=100%

Comments (Including reason for exclusion)

________________________________________________________________________________________________________________________________________________________________________________________________________________________________________________________________________________________________

JBI Critical Appraisal Checklist for
analytical cross sectional studies

Reviewer __A. Ntunja_________Date__01 May 2024_________

Author_____ Ben Jmaa et al., 2023____ Year___2023______ Record Number__5______

|  | Yes | No | Unclear | Not applicable |
| --- | --- | --- | --- | --- |
| 1. Were the criteria for inclusion in the sample clearly defined? | x | □ | □ | □ |
| 1. Were the study subjects and the setting described in detail? | x | □ | □ | □ |
| 1. Was the exposure measured in a valid and reliable way? | x | □ | □ | □ |
| 1. Were objective, standard criteria used for measurement of the condition? | x | □ | □ | □ |
| 1. Were confounding factors identified? |  | x | □ | □ |
| 1. Were strategies to deal with confounding factors stated? |  | x | □ | □ |
| 1. Were the outcomes measured in a valid and reliable way? | x | □ | □ | □ |
| 1. Was appropriate statistical analysis used? | x | □ | □ | □ |

Overall appraisal: Include x Exclude □ Seek further info □. 6/8= 75%

Comments (Including reason for exclusion)

________________________________________________________________________________________________________________________________________________________________________________________________________________________________________________________________________________________________

JBI Critical Appraisal Checklist for
analytical cross sectional studies

Reviewer __A. Ntunja_________Date__01 May 2024_________

Author_____ AbouZeid et al. (2022)____ Year___2022______ Record Number__6______

|  | Yes | No | Unclear | Not applicable |
| --- | --- | --- | --- | --- |
| 1. Were the criteria for inclusion in the sample clearly defined? | x | □ | □ | □ |
| 1. Were the study subjects and the setting described in detail? | x | □ | □ | □ |
| 1. Was the exposure measured in a valid and reliable way? | x | □ | □ | □ |
| 1. Were objective, standard criteria used for measurement of the condition? | x | □ | □ | □ |
| 1. Were confounding factors identified? | x | x | □ | □ |
| 1. Were strategies to deal with confounding factors stated? |  | x | □ | □ |
| 1. Were the outcomes measured in a valid and reliable way? | x | □ | □ | □ |
| 1. Was appropriate statistical analysis used? | x | □ | □ | □ |

Overall appraisal: Include x Exclude □ Seek further info □ 7/8=87.5%

Comments (Including reason for exclusion)

_______________________________________________________________________________________________________________________________________________________________________________________________________________________________________________________________________________________________

JBI Critical Appraisal Checklist for
analytical cross sectional studies

Reviewer __A. Ntunja_________Date__01 May 2024_________

Author_____ Millanzi et al. (2023)____ Year___2023______ Record Number__7______

|  | Yes | No | Unclear | Not applicable |
| --- | --- | --- | --- | --- |
| 1. Were the criteria for inclusion in the sample clearly defined? | x | □ | □ | □ |
| 1. Were the study subjects and the setting described in detail? | x | □ | □ | □ |
| 1. Was the exposure measured in a valid and reliable way? | x | □ | □ | □ |
| 1. Were objective, standard criteria used for measurement of the condition? | x | □ | □ | □ |
| 1. Were confounding factors identified? | x |  | □ | □ |
| 1. Were strategies to deal with confounding factors stated? |  |  | x | □ |
| 1. Were the outcomes measured in a valid and reliable way? | x | □ | □ | □ |
| 1. Was appropriate statistical analysis used? | x | □ | □ | □ |

Overall appraisal: Include x Exclude □ Seek further info□ 7/8=87.5%

Comments (Including reason for exclusion________________________________________________________________________________________________________________________________________________________________________________________________________________________________________________________________________________________

JBI Critical Appraisal Checklist for
analytical cross sectional studies

Reviewer __A. Ntunja_________Date__01 May 2024_________

Author_____ Onoh et al. ( 2019)____ Year___2019______ Record Number__8______

|  | Yes | No | Unclear | Not applicable |
| --- | --- | --- | --- | --- |
| 1. Were the criteria for inclusion in the sample clearly defined? | x | □ | □ | □ |
| 1. Were the study subjects and the setting described in detail? | x | □ | □ | □ |
| 1. Was the exposure measured in a valid and reliable way? | x | □ | □ | □ |
| 1. Were objective, standard criteria used for measurement of the condition? | x | □ | □ | □ |
| 1. Were confounding factors identified? |  | x | □ | □ |
| 1. Were strategies to deal with confounding factors stated? |  |  |  | x |
| 1. Were the outcomes measured in a valid and reliable way? | x | □ | □ | □ |
| 1. Was appropriate statistical analysis used? | x | □ | □ | □ |

Overall appraisal: Include x Exclude □ Seek further info □. 6/8= 75%

Comments (Including reason for exclusion)

________________________________________________________________________________________________________________________________________________________________________________________________________________________________________________________________________________________________

JBI Critical Appraisal Checklist for
analytical cross sectional studies

Reviewer __A. Ntunja_________Date__01 May 2024_________

Author_____ Sarker et al. (2014) ____ Year___2014______ Record Number__9______

|  | Yes | No | Unclear | Not applicable |
| --- | --- | --- | --- | --- |
| 1. Were the criteria for inclusion in the sample clearly defined? | x | □ | □ | □ |
| 1. Were the study subjects and the setting described in detail? | x | □ | □ | □ |
| 1. Was the exposure measured in a valid and reliable way? |  | □ | □ | □x |
| 1. Were objective, standard criteria used for measurement of the condition? | x | □ | □ | □ |
| 1. Were confounding factors identified? | x |  | □ | □ |
| 1. Were strategies to deal with confounding factors stated? | x |  |  |  |
| 1. Were the outcomes measured in a valid and reliable way? | x | □ | □ | □ |
| 1. Was appropriate statistical analysis used? | x | □ | □ | □ |

Overall appraisal: Include x Exclude □ Seek further info □. 7/8= 87.5%

Comments (Including reason for exclusion)

________________________________________________________________________________________________________________________________________________________________________________________________________________________________________________________________________________________________

JBI Critical Appraisal Checklist for
analytical cross sectional studies

Reviewer __A. Ntunja_________Date__01 May 2024_________

Author_____ Tabash et al. (2016) ____ Year___2016______ Record Number__10______

|  | Yes | No | Unclear | Not applicable |
| --- | --- | --- | --- | --- |
| 1. Were the criteria for inclusion in the sample clearly defined? | x | □ | □ | □ |
| 1. Were the study subjects and the setting described in detail? | x | □ | □ | □ |
| 1. Was the exposure measured in a valid and reliable way? | x | □ | □ | □ |
| 1. Were objective, standard criteria used for measurement of the condition? | x | □ | □ | □ |
| 1. Were confounding factors identified? |  |  | x | □ |
| 1. Were strategies to deal with confounding factors stated? |  |  | x |  |
| 1. Were the outcomes measured in a valid and reliable way? | x | □ | □ | □ |
| 1. Was appropriate statistical analysis used? | x | □ | □ | □ |

Overall appraisal: Include x Exclude □ Seek further info □. 6/8= 75%

Comments (Including reason for exclusion)

________________________________________________________________________________________________________________________________________________________________________________________________________________________________________________________________________________________________

JBI Critical Appraisal Checklist for
analytical cross sectional studies

Reviewer __A. Ntunja_________Date__01 May 2024_________

Author_____ Sapkota et al. (2014) ____ Year___2014______ Record Number__11______

|  | Yes | No | Unclear | Not applicable |
| --- | --- | --- | --- | --- |
| 1. Were the criteria for inclusion in the sample clearly defined? |  | x | □ | □ |
| 1. Were the study subjects and the setting described in detail? | x | □ | □ | □ |
| 1. Was the exposure measured in a valid and reliable way? | x | □ | □ | □ |
| 1. Were objective, standard criteria used for measurement of the condition? | x | □ | □ | □ |
| 1. Were confounding factors identified? |  |  | x | □ |
| 1. Were strategies to deal with confounding factors stated? |  |  | x |  |
| 1. Were the outcomes measured in a valid and reliable way? | x | □ | □ | □ |
| 1. Was appropriate statistical analysis used? | x | □ | □ | □ |

Overall appraisal: Include x Exclude □ Seek further info □. 5/8= 62.5%

Comments (Including reason for exclusion)

________________________________________________________________________________________________________________________________________________________________________________________________________________________________________________________________________________________________

JBI Critical Appraisal Checklist for
analytical cross sectional studies

Reviewer __A. Ntunja_________Date__01 May 2024_________

Author_____ Kumar et al. (2016 ) ____ Year___2016______ Record Number__12______

|  | Yes | No | Unclear | Not applicable |
| --- | --- | --- | --- | --- |
| 1. Were the criteria for inclusion in the sample clearly defined? |  | x | □ | □ |
| 1. Were the study subjects and the setting described in detail? | x | □ | □ | □ |
| 1. Was the exposure measured in a valid and reliable way? | x | □ | □ | □ |
| 1. Were objective, standard criteria used for measurement of the condition? | x | □ | □ | □ |
| 1. Were confounding factors identified? |  |  | x | □ |
| 1. Were strategies to deal with confounding factors stated? |  |  |  | X |
| 1. Were the outcomes measured in a valid and reliable way? | x | □ | □ | □ |
| 1. Was appropriate statistical analysis used? | x | □ | □ | □ |

Overall appraisal: Include x Exclude □ Seek further info □. 5/8= 62.5%

Comments (Including reason for exclusion)

________________________________________________________________________________________________________________________________________________________________________________________________________________________________________________________________________________________________

JBI Critical Appraisal Checklist for
analytical cross sectional studies

Reviewer __A. Ntunja_________Date__01 May 2024_________

Author_____ Basavaraj et al. (2021) ____ Year____2021_____ Record Number__13______

|  | Yes | No | Unclear | Not applicable |
| --- | --- | --- | --- | --- |
| 1. Were the criteria for inclusion in the sample clearly defined? | x |  | □ | □ |
| 1. Were the study subjects and the setting described in detail? | x | □ | □ | □ |
| 1. Was the exposure measured in a valid and reliable way? |  | □ | □ | x |
| 1. Were objective, standard criteria used for measurement of the condition? | x | □ | □ | □ |
| 1. Were confounding factors identified? |  | x |  | □ |
| 1. Were strategies to deal with confounding factors stated? |  |  |  | X |
| 1. Were the outcomes measured in a valid and reliable way? | x | □ | □ | □ |
| 1. Was appropriate statistical analysis used? | x | □ | □ | □ |

Overall appraisal: Include x Exclude □ Seek further info □. 5/8= 62.5%

Comments (Including reason for exclusion)

________________________________________________________________________________________________________________________________________________________________________________________________________________________________________________________________________________________________

JBI Critical Appraisal Checklist for
analytical cross sectional studies

Reviewer __A. Ntunja_________Date__01 May 2024_________

Author_____ Melaku & Tiruneh____ Year___2020______ Record Number__14______

|  | Yes | No | Unclear | Not applicable |
| --- | --- | --- | --- | --- |
| 1. Were the criteria for inclusion in the sample clearly defined? | x |  | □ | □ |
| 1. Were the study subjects and the setting described in detail? | x | □ | □ | □ |
| 1. Was the exposure measured in a valid and reliable way? |  | □ | □ | x |
| 1. Were objective, standard criteria used for measurement of the condition? | x | □ | □ | □ |
| 1. Were confounding factors identified? | X |  |  | □ |
| 1. Were strategies to deal with confounding factors stated? |  | X |  |  |
| 1. Were the outcomes measured in a valid and reliable way? | x | □ | □ | □ |
| 1. Was appropriate statistical analysis used? | x | □ | □ | □ |

Overall appraisal: Include x Exclude □ Seek further info □. 6/8= 75%

Comments (Including reason for exclusion)

________________________________________________________________________________________________________________________________________________________________________________________________________________________________________________________________________________________________

JBI Critical Appraisal Checklist for
analytical cross sectional studies

Reviewer __A. Ntunja_________Date__01 May 2024_________

Author_____ Temesgen et al. (2022)____ Year___2022______ Record Number__15______

|  | Yes | No | Unclear | Not applicable |
| --- | --- | --- | --- | --- |
| 1. Were the criteria for inclusion in the sample clearly defined? | x |  | □ | □ |
| 1. Were the study subjects and the setting described in detail? | x | □ | □ | □ |
| 1. Was the exposure measured in a valid and reliable way? |  | □ | □ | x |
| 1. Were objective, standard criteria used for measurement of the condition? | x | □ | □ | □ |
| 1. Were confounding factors identified? | X |  |  | □ |
| 1. Were strategies to deal with confounding factors stated? | X |  |  |  |
| 1. Were the outcomes measured in a valid and reliable way? | x | □ | □ | □ |
| 1. Was appropriate statistical analysis used? | x | □ | □ | □ |

Overall appraisal: Include x Exclude □ Seek further info □. 7/8= 87.5%

Comments (Including reason for exclusion)

________________________________________________________________________________________________________________________________________________________________________________________________________________________________________________________________________________________________
